# Examining interactions of illness perceptions, avoidance behavior and patient status in predicting quality of life among people with irritable bowel syndrome

**DOI:** 10.1080/21642850.2024.2311986

**Published:** 2024-02-05

**Authors:** Malin Ekholm, Marit Krouwels, Keegan Knittle

**Affiliations:** a Faculty of Social Sciences, University of Helsinki, Helsinki, Finland; b Faculty of Sport and Health Sciences, University of Jyväskylä, Jyväskylä, Finland; c Psychotherapie en Diagnostiek Leiden (Independent Psychology Practice), Leiden, Netherlands

**Keywords:** Irritable bowel syndrome, coping behavior, avoidance behavior, illness perceptions, treatment-seeking behavior

## Abstract

**Background:** Illness perceptions (IPs) and avoidance behavior both predict quality of life (QoL) in people with irritable bowel syndrome (IBS). This study examined whether the effects of IPs on QoL are mediated by avoidance behaviors, and whether this mediation is moderated by participant treatment-seeking status.

**Methods:** People with self-reported IBS (*n* = 253) answered a survey assessing QoL, IPs, avoidance behaviors, and treatment-seeking status. Moderated-mediation analyses investigated the paths from IPs through avoidance behaviors to QoL, with treatment-seeking status entered as a moderator.

**Results:** The final moderated mediation model included the IPs *consequences*, *timeline* and *emotional representations* as independent variables and avoidance behavior and depressive reactions as mediators. This model explained 68.6% of the variance in QoL. Among treatment-seeking participants five significant mediation effects were found, whereas only one significant mediation effect was found among participants who did not report seeking treatment.

**Conclusions:** IPs seem to drive avoidant behavioral responses to IBS symptoms, which in turn predict reductions in QoL. These relationships seem more pronounced among people who seek treatment for their symptoms. In practice, health care practitioners might help improve the QoL of people with IBS by preventing or remedying the development of negative IPs and avoidance behaviors.

## Introduction

Irritable Bowel Syndrome (IBS) is a chronic functional gastrointestinal disorder associated with discomfort, abdominal pain and changes in bowel movements. IBS is one of the most common bowel disorders with a global prevalence rate of 9.2%, which varies widely across countries (1.1%–45%) (Lovell & Ford, 2012; Oka et al., 2020). IBS is also considerably more common for women than men; women being twice as likely to meet IBS criteria (Drossman et al., 1993). IBS accounts for an estimated 2.4–3.5 million physician visits per year in the US and 2.2 million medication prescriptions; costing the healthcare system considerably more than healthy individuals (Akehurst et al., 2002; Drossman et al., 1997). Patients with IBS also report lower quality of life, and more time off work due to their condition (Akehurst et al., 2002). Given both the socioeconomic impact and wellbeing effects, it is important to understand factors that may improve quality of life among people suffering from IBS.

IBS can be diagnosed according to the following Rome-III criteria: recurrent abdominal pain or discomfort at least three days per month in the last three months, which is associated with two of the following: improvement with defecation, change in frequency of stool and/or change in form of stool. Additionally, four subtypes of IBS can be distinguished: IBS-D, IBS-C, IBS-M and IBS-U. Each subtype is characterized by predominant complaints; diarrhea for IBS-D, constipation for IBS-C, mixed complaints for IBS-M and unspecified complaints for IBS-U (Longstreth et al., 2006).

While diagnostics have made progress, IBS remains a complex and poorly understood condition with no clear etiology. Current perspectives however agree on the biopsychosocial nature of the disorder, i.e. IBS and its associated symptoms are a result of the interaction between psychosocial and biological factors (Hauser et al., 2014; Lutgendorf & Costanzo, 2003). IBS can also be understood as a disordered signaling between the central nervous system (the brain) and the gastrointestinal system (the gut), with different irregularities on the gut-brain axis. These irregularities include changes in gut motility, visceral sensitivity and altered gut microbiome (Simrén & Tack, 2018). According to the biopsychosocial model for functional gastrointestinal disorders, psychosocial factors, life events, trauma or stress can disrupt the signaling between the brain and the gut, causing the symptoms of IBS (Lackner et al., 2004). Studies have also demonstrated a high prevalence of affective disorders in the IBS populations, which emphasizes the role of psychosocial factors in understanding the condition. For example, up to 34% of IBS patients meet the criteria for Generalized Anxiety Disorder (GAD), and 37% of patients with GAD meet the criteria for IBS (Drews & Hazlett-Stevens, 2008). Evidence suggests that anxiety and depression can act as predictors of IBS and coping behavior – indicating a significant overlap between affective disorders and IBS (Hauser et al., 2014; Labus et al., 2007).

To cope with IBS and its symptoms, patients often deploy avoidant coping strategies, or avoidance behaviors. These behaviors are commonly associated with anxiety over symptoms and attempts to control these (David et al., 2021; Labus et al., 2007). According to LeDoux et al. (2017) the development and maintenance of avoidant behavior is associated with perceiving certain stimuli as threatening or aversive, and these avoidant responses are reinforced through operant conditioning. In the case of IBS, patients may avoid going out, due to worrying that they might experience bowel symptoms or problems. By avoiding the feared situation and the anxiety associated with it, this behavior is then reinforced. This strategy might become persistent and habitual, preventing the patient from building more proactive strategies to cope with the distress caused by feared symptoms. Avoidant coping strategies might thereby help the individual to stay in control in the short term, but avoidance behaviors can also act as a maintaining mechanism for IBS (Bonnert et al., 2018; Jones et al., 2006; Keefer et al., 2005). Avoidant coping is associated with depressive symptoms, anxiety, quality of life and symptom severity, which in turn may further increase avoidance behaviors (Crane & Martin, 2004; David et al., 2021; Labus et al., 2007; Torkzadeh et al., 2019). Symptom anxiety and avoidant coping strategies can thereby create a vicious cycle, which increases the extent of avoidance behavior.

Given the role of behavior in IBS, a further look into the antecedents of coping responses is warranted. According to the cognitive–behavioral model, IBS symptoms are affected and maintained by how patients cognitively react to gastrointestinal symptoms and life-events. These cognitive reactions will in turn shape the emotional responses (e.g. anxiety), severity of symptoms and coping behavior (Kennedy et al., 2012; Toner et al., 1998). The Common-Sense Model of Self-Regulation (CSM; Leventhal et al., 2016) proposes a similar mechanism. The CSM suggests that behavioral responses to illness are a result of cognitive and emotional representations people construct of their illness or condition. According to the CSM, patients form representations (or illness perceptions) around five different dimensions: identity, causal attributions, expectations regarding duration, perceived consequences, and perceived control over illness. These representations have both a direct effect on wellbeing outcomes, as well as an indirect effect mediated by coping responses (Hagger et al., 2017). In the context of IBS, illness perceptions have been shown to predict various outcomes, including quality of life, depression and anxiety, as well as treatment effects in cognitive–behavioral interventions (Chilcot & Moss-Morris, 2013; David et al., 2021; Rutter & Rutter, 2007). Perceived consequences of IBS, perceived personal or treatment control, as well as emotional representations are also associated with treatment-seeking behavior and healthcare use (Schwille-Kiuntke et al., 2021).

Around 30% of people who experience symptoms of IBS consult a physician, meaning that a vast majority do not seek treatment. A distinction is often made between treatment-seeking patients (IBS patients) and people who do not seek treatment (IBS non-patients), and evidence suggests that these groups differ from each other in various ways. While studies have found that there are no differences in gastrointestinal symptoms in the two groups, IBS patients tend to report higher pain scores, poorer coping resources, greater levels of anxiety and psychological symptoms, and lower quality of life (Canavan et al., 2014; Ringström et al., 2007). IBS patients also tend to perceive their IBS as having greater consequences on their daily lives (Schwille-Kiuntke et al., 2021). There is, however, some evidence suggesting no differences between patients and non-patients in personality traits, interpersonal distress, and temporary psychological distress (Weinryb et al., 2003). Further research on patient status and its effects is thereby needed to better understand its role in predicting outcomes in IBS.

To further understand the interplay between cognitive and behavioral factors in IBS, this study aims to examine the relationships between illness perceptions, avoidance behavior and quality of life in people with IBS symptoms, and in particular, to examine whether treatment-seeking behavior moderates these relationships.

## Materials and methods

### Participants and procedures

Participants in this non-experimental cross-sectional study were recruited in the spring of 2009 through websites of Dutch IBS patient organizations (www.pdsb.nl and www.mlds.nl), through IBS social media groups and by word of mouth. Messages advertising the study were placed on to these websites and social media groups and contained a link to an online survey hosted on www.surveymonkey.com. The messages also indicated that participants in the study could choose to enter a prize drawing for a €100 gift card.

Participants first chose the language (Dutch or English) for the questionnaire, and then received additional information about the study and contact details for the research team. Before beginning the survey, participants needed to provide their informed consent, affirm that they were at least 18 years of age and self-report having IBS. Completing all study measures took around 15 min, and, at the end of the survey, participants could voluntarily provide their contact details to enter the €100 prize draw.

All study procedures were conducted in line with the Declaration of Helsinki; however, this study did not undergo full review by an institutional review board or ethical committee. This study was conducted as part of a Master’s thesis project and its methods were reviewed by senior academics and the head of the Health Psychology department of Leiden University prior to the start of the study. Given the non-interventional nature of the study and the minimal burden and risks it presented to participants, it was concluded that the study could proceed without full review from an ethical committee. This was later confirmed by the Human Sciences Ethics Committee of the University of Jyväskylä, in line with the guidelines of the Finnish National Board on Research Integrity (2019).

### Measures

#### Demographic characteristics

Participants provided demographic information including age, gender, marital status and employment status, and indicated how they had heard about the study.

#### IBS status

The IBS module of the ROME III questionnaire (Drossman et al., 2006) was used to assess whether participants met the ROME III criteria for IBS. This 10-item self-report questionnaire contains items assessing the frequency of recurrent abdominal pain or discomfort, time since onset of recurring abdominal pain or discomfort, and relationships between recurring abdominal pain or discomfort and changes in the frequency and form of bowel movements. Responses to items are fed into an algorithm which can identify whether a person meets the ROME III diagnostic criteria for IBS, namely, recurrent abdominal pain and/or discomfort at least 3 days per month for at least 3 months, with onset at least 6 months previously. This pain or discomfort should additionally be associated with two or more of the following: improvement with defecation, onset associated with a change in frequency of stool, or onset associated with a change in appearance of stool. The algorithm can further distinguish between the IBS subtypes IBS-C, IBS-D, IBS-M and IBS-U. After collection of the data for this study, the ROME III criteria were replaced by the ROME IV version, in which the term ‘discomfort’ was removed and the required frequency of symptoms has been increased; thereby emphasizing the experiences of pain and more frequent symptoms as the criteria for diagnosis (Lacy et al., 2016).

As background information, participants also stated how long they had suffered from IBS symptoms, and whether they had been diagnosed with any other gastrointestinal disorders that might explain their symptoms.

#### IBS-related treatment-seeking behavior

Treatment-seeking behavior was assessed in a method similar to that used by Jemilohun et al. (2018). A ‘yes or no’ question asked participants whether a doctor had diagnosed them with IBS. Receiving this clinical diagnosis was assumed to constitute having sought treatment for IBS-related symptoms.

#### Illness perceptions

Illness perceptions were assessed with the Revised Illness Perception Questionnaire (IPQ-R; Moss-Morris et al., 2002). The IPQ-R consists of 38 items assessing illness representations according to Leventhal’s CSM (Leventhal et al., 2016), through the dimensions of identity, consequences, timeline acute/chronic, timeline cyclical, illness coherence and emotional. The identity and causal dimensions of the scale were not administered to participants in this study, as the symptoms of IBS are inherent in the ROME III diagnostic criteria.

#### Avoidance behavior and avoidant coping

Subscales from multiple questionnaires were used to assess avoidance behavior. These include the 7-item ‘interference with activity’ subscale from the IBS-QOL (Patrick et al., 1998) and the ‘depressive reactions’ (6 items), ‘palliative reactions’ (6 items) and ‘avoidance’ (8 items) subscales from the Utrechtse Coping Lijst (UCL; Schreurs et al., 1988). The UCL items are responded to on a four-point Likert scale, with higher scores indicating a more frequent/likely response to a stressful situation. Respondents were additionally asked a yes or no question about whether they avoid specific places or situations due to their IBS symptoms. If they answered yes, then a free response question allowed participants to list the places or situations they typically avoid.

#### IBS quality of life

The IBS-QOL questionnaire (Patrick et al., 1998) was used to assess IBS-specific quality of life. It contains 34 items with responses on a 5-point Likert scale. The questionnaire yields eight different subscales (dysphoria, interference with activity, body image, health worry, food avoidance, social reaction, sexual, relationships) and an overall IBS-specific quality of life score. Scores on the IBS-QoL range from 0–100, with higher scores indicating lower quality of life. We used the ‘interference with activity’ subscale as a measure of avoidance behavior, due to its nonoverlap with other avoidance behavior measures used in this study. Therefore, the IBS-QOL total score has been calculated based on only the remaining seven QoL categories (i.e. not including the ‘interference with activity’ scale).

## Design and data analysis

We used SPSS to apply the ROME III IBS diagnostic criteria and calculate the subscales of the questionnaires under study. Then, ANOVAs explored possible differences in IBSQoL across different levels of demographic variables. We next examined bivariate correlations between illness perceptions, avoidance behaviors and IBS quality of life. Moderated mediation models were conducted using the medmod R package (https://blog.jamovi.org/2017/09/25/medmod.html) in JAMOVI. These moderated mediation models included illness perceptions as independent variables, self-reported avoidance behavior and avoidant coping strategies as mediators, and IBS QOL as the dependent variable. IBS-related treatment-seeking behavior (seeking tretment or not seeking treatment) was entered as a moderator variable. We ran an initial model that included all variables. We then created a simplified model that only included variables with significant a or b paths in the full model. This yielded a more parsimonious final model (Falk & Muthukrishna, 2023). All models were run twice: Once including all participants and a second time excluding participants who did not meet the Rome III criteria. The data files and syntax needed to reproduce all analyses are available on the project’s Open Science Framework page (https://osf.io/cwkfq/).

## Results

A total of 253 individuals with self-reported IBS provided informed consent and completed the questionnaire. The sample was 84% female and had a mean age of 34 years (SD = 12.6). Of these participants, 158 (62%) met the Rome III criteria for IBS according to the Rome III questionnaire, whereas 95 (38%) did not. One-hundred seventy four participants (69%) reported being either married (*n *= 83, 33%) or in a relationship (*n *= 91, 36%), while 73 (29%) were single. There were no significant differences in IBS-QoL across levels of any of these demographic variables, and no significant correlation between age and IBS-QoL (all *p*>.05; Results available in the supplement).

Of these participants, 122 (48.6%) reported avoiding specific situations or activities due to (worries about) their bowel problems. As shown in Table 1, the most commonly reported situations and activities were different types of leisure time activities (*n *= 64, 52%), social situations (*n *= 53, 43%) and places or situations with unknown access to the bathroom (*n *= 37, 30%).

**Table 1.** Situations and activities that participants reported avoiding due to their IBS symptoms.

Reportedly avoided situation or activity	n (%)
Leisure time activities	64 (52)
Social situations	53 (43)
Places or situations with unknown access to the bathroom	37 (30)
Eating in public	27 (22)
Transport or traveling	21 (17)
Work or school	6 (5)
Romantic relationships	2 (2)

In examining bivariate correlations between the variables under study, all IP subscales except for timeline were significantly correlated with IBS QoL. Additionally, all IP variables were significantly correlated with at least one coping behavior. Avoidance behavior, avoidant coping and depressive reactions were correlated with IBS QoL, but palliative reactions were not. These correlations are presented in Table 2.

**Table 2.** Means and standard deviations of included variables, as well as correlations between them.

Variables	M	SD	1	2	3	4	5	6	7	8	9	10	11	12
1. IBS-Qol	32.3	19.78	-											
2. Avoidance behavior	34.4	1.49	0.76***											
3. Depressive reactions (UCL)	11.8	0.20	0.50***	0.36***										
4. Palliative reactions (UCL)	14.1	0.21	0.12	0.08	0.06									
5. Avoidance (UCL)	17.6	0.24	0.33**	0.31***	0.43***	0.20**								
6. Timeline	22.6	4.15	0.34***	0.38***	0.06	0.11	0.15*							
7. Timecycle	14.8	3.08	−0.03	−0.01	0.00	0.05	0.08	0.05						
8. Consequences	18.1	5.03	0.66***	0.64***	0.44***	−0.02	0.27***	0.31***	0.02					
9. Personal control	17.2	3.61	−0.18**	−0.20**	−0.04	0.04	−0.027	−0.242***	0.09	−0.186				
10. Treatment control	14.2	3.30	−0.17*	−0.24***	−0.03	0.08	−0.03	−0.421***	−0.02	−0.118	0.35***			
11. Illness coherence	14.8	4.09	−0.32***	−0.21**	−0.25***	0.05	−0.13	−0.004	−0.26***	−0.38***	0.26***	0.08		
12. Emotional representations	17.5	4.98	0.66***	0.49***	0.49***	0.08	0.23***	0.10	0.01	0.64***	−0.07	−0.05	−0.4***	

*p < 0.05 **p < 0.01 ***p <0.001.

### Moderated mediation results

**Figure 1.**
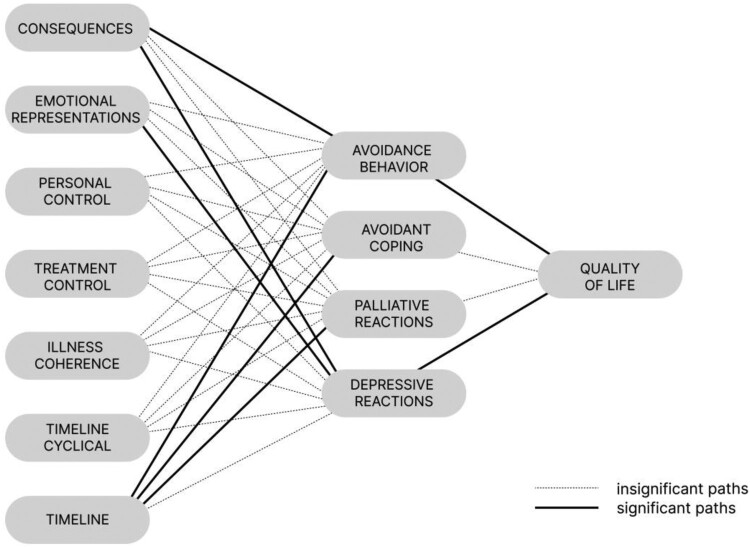
Full mediation model including all variables under study. Solid lines indicate significant paths and dotted lines indicate insignificant paths.

Initially, all seven illness perception variables were entered as independent variables, self-reported avoidance behavior and three avoidant coping strategies were entered as mediators, IBS QOL was used as the dependent variable (see Figure 1) and treatment-seeking behavior was entered as the moderator. In this full model, six out of the 28 investigated a paths (i.e. between illness perceptions and avoidance behavior) were significant. Similarly, two out of the four investigated b paths (i.e. between coping responses and IBS quality of life) were significant. Two of the *a × b* paths were significant (emotional representations through depressive reactions to IBSQoL, and consequences through avoidance behavior to IBSQoL). This initial model explained 67.9% of the variance in IBSQoL (adjusted R^2^), and full results are shown in the supplementary material. Variables in this full model from which significant *a* or *b* paths arose were selected for inclusion into a final simplified moderated mediation model. These variables were the illness perceptions (independent variables) consequence, emotional representations and timeline and the coping behaviors (mediators) avoidant behavior and depressive reactions.

**Figure 2.**
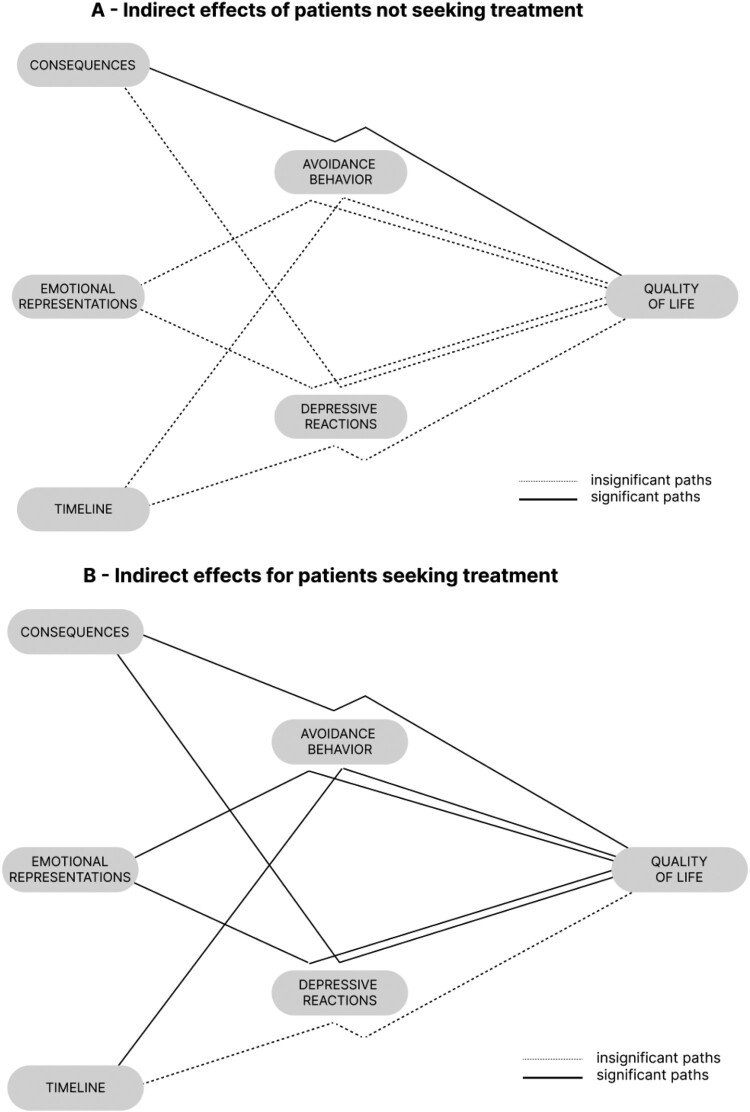
Simplified mediation analyses, which exclude variables not significantly related to quality of life. Models are presented separately for participants who reported seeking treatment for their IBS symptoms (Panel B), and for participants who did not report seeking treatment for their IBS symptoms (Panel A). Solid lines indicate significant indirect paths and dotted lines indicate insignificant indirect paths.

The simplified moderated mediation model is shown in Figure 2. This simplified model fit the data better than the full model (adjusted *R*^2^ = 68.6%), and revealed two significant indirect paths. When examining the moderation effect of treatment-seeking behavior, one of these indirect paths was significant among participants who did not report seeking treatment (Figure 2, Panel A), while five were significant among participants who did report treatment-seeking behavior (Figure 2, Panel B). In other words, the indirect relationships from illness perceptions through behavioral responses to IBSQoL were stronger for participants who reported seeking treatment for their IBS symptoms. Post hoc power analyses indicated that this model had 99% power to detect significant a or b paths of magnitude *r = *0.39, and 94% power to detect significant moderator effects of magnitude *d *= 0.5 at the alpha = .05 level. Full results of the simplified moderated mediation models are shown in Table 3.

**Table 3.** Results of simplified moderated mediation models.

Moderator	IV	a path	95% C.I.	Mediator	b path	95% C.I.	DV	ab path	95% C.I.
Treatment-seeking	Consequences	2.12***	1.45–2.79	Avoidance behavior	0.37***	0.28–0.45	IBS – QoL	0.78***	0.47–1.08
	Consequences	0.14*	0.03–0.25	Depressive reactions	0.095***	0.44–1.47	IBS – QoL	0.13*	0.01–0.26
	Emotional representations	1.08***	0.47–1.72	Avoidance behavior	0.37***	0.28–0.45	IBS – QoL	0.40**	0.15–0.65
	Emotional representations	0.26***	0.15–0.36	Depressive reactions	0.095***	0.44–1.47	IBS – QoL	0.24**	0.08–0.41
	Timeline	1.51***	0.83–2.19	Avoidance behavior	0.37***	0.28–0.45	IBS – QoL	0.55***	0.27–0.83
	Timeline	0.02	−0.09–0.13	Depressive reactions	0.095***	0.44–1.47	IBS – QoL	0.02	−0.08–0.13
Not seeking treatment	Consequences	2.26**	0.88–3.64	Avoidance behavior	0.28***	0.20–0.38	IBS – QoL	0.64*	0.21–1.08
	Consequences	0.18	−0.04–0.41	Depressive reactions	0.75**	0.23–1.26	IBS – QoL	0.14	−0.05–0.33
	Emotional representations	0.02	−1.39–1.43	Avoidance behavior	0.28***	0.20–0.38	IBS – QoL	0.01	−0.40–0.41
	Emotional representations	0.17	−0.06–0.40	Depressive reactions	0.75**	0.23–1.26	IBS – QoL	0.13	−0.06–0.32
	Timeline	0.65	−0.35–0.00	Avoidance behavior	0.28***	0.20–0.38	IBS – QoL	0.19	−0.13–0.50
	Timeline	−0.18	−0.45–1.76	Depressive reactions	0.75**	0.23–1.26	IBS – QoL	−0.13	−0.29–0.03

* *p* < 0.05 **p < 0.01 *** *p* < 0.001.

### Sensitivity analyses

As this study included a self-selected sample of people who reported having IBS, the analyses were repeated including only participants who met the ROME III criteria. The results of these analyses are available in the supplementary material. These models yielded largely the same pattern of results. In the initial model, five out of the 28 investigated a-paths (i.e. between illness perceptions and avoidance behavior) were significant and two of the b-paths (i.e. between coping responses and IBS quality of life) were significant. One of the a × b paths were significant (consequence through avoidance behavior to IBSQoL). When examining the moderation effect of treatment-seeking behavior, none of the indirect paths were significant among participants who did not report seeking treatment, while three remained significant among participants who did report seeking treatment. Conclusions thereby remain the same; the indirect relationships from perceptions through behavioral responses to IBS-QoL was stronger for the respondents who had sought treatment.

## Discussion

This study explored the roles of illness perceptions and avoidance behaviors in predicting health-related quality of life among people with self-reported irritable bowel syndrome. Consequences, timeline and emotional representations were most strongly related to the behavioral coping strategies reported by participants. People with more negative perceived consequences of IBS, negative emotional representations and longer perceived duration (timeline) of IBS reported more avoidant and depressive coping. This was in turn associated with lower reported quality of life. The results further show that these relationships were moderated by IBS patient status, in that the relationship is stronger for patients who reported seeking treatment.

These findings are congruent with the mechanisms proposed by the Common-Sense Model of Self-Regulation (Hagger et al., 2017; Leventhal et al., 2016), and the results are similar to those of previous studies investigating the role of illness perceptions in IBS. Several earlier studies have found that emotional representations and perceived consequences predict QoL, and that this relationship is mediated by coping responses (e.g. Knowles et al., 2017; Rutter & Rutter, 2002). To our knowledge, expectations of duration (timeline) of IBS has however not previously been linked to avoidant coping – providing a new perspective into the development and maintenance of these behaviors.

This study did not find significant relationships between other illness perceptions, avoidance behavior and IBS-QoL. The correlation analysis did however find that positive perceptions of control were significantly correlated with avoidance behavior and quality of life. This correlation was negative, meaning that stronger control beliefs correlated with less maladaptive coping and better quality of life. The correlations were not strong, and we did not find any significant paths between control beliefs, avoidance behavior and quality of life. Control beliefs have, however, been found to be significantly associated with psychological outcomes and treatment-seeking behavior. Rutter and Rutter (2007) found that stronger control beliefs were associated with better quality of life, satisfaction with health, and less anxiety. Schwille-Kiuntke et al. (2021) also found that patients who use medication for their IBS have weaker perceived control than those who do not, suggesting that weaker control beliefs might be correlated with treatment-seeking behaviors.

Similarly to previous studies, avoidance behaviors were also prevalent in this sample, with almost half reporting avoiding certain places or situations due to their IBS. Of the behavioral responses assessed in this study, only avoidance behavior (IBS-QoL interference with activity – subscale) and depressive reactions seemed related to quality of life. These results are similar to those found in previous studies also linking avoidant and emotion-focused coping to worse health outcomes (David et al., 2021). Torkzadeh et al. (2019) also found a relationship between palliative coping strategies and worsened quality of life, but this relationship was not found in this study.

To assess coping behaviors, this study used subscales from both the IBS-QoL and the UCL. While the IBS-QoL scale is developed specifically for patients with IBS, the UCL is not widely used in this population. It should also be noted that the scales used to measure coping behavior vary greatly across studies, which makes comparisons and accumulation of evidence difficult. In a review investigating coping behavior in IBS, David et al. (2021) identified 15 different tools to assess coping in only 21 included studies, and found inconclusive results regarding the effects of active coping strategies. Future studies should further investigate the effects of different coping strategies and aim to consolidate the numerous ways to measure coping, e.g. through establishing clear standards for assessing coping behaviors in the IBS population.

To our knowledge, no previous studies have investigated the moderating effects of treatment-seeking behavior, i.e. patient status, on the relationships between illness perceptions, behavior and quality of life in IBS. While previous studies have found differences in illness perceptions, psychosocial wellbeing and quality of life between IBS patients and non-patients (Ringström et al., 2007; Schwille-Kiuntke et al., 2021), the relationship between these factors and behavior had not yet been explored. The results of this study show that a diagnosis seems to be linked with avoidance behavior and worse quality of life. In practice, this means that it is important to understand ways to prevent people with IBS from developing maladaptive coping styles. Upon diagnosis, health care practitioners (HCPs) should discuss the possible role of avoidance behavior with their IBS patients. These discussions should focus on how to prevent the development of avoidance behavior and thereby worsened quality of life. Previous studies have identified several techniques associated with improved quality of life in IBS patients, which practitioners can incorporate into their interactions with patients. These include self-monitoring of cognitions, coping planning, and supportive non-directive discussions about symptoms and patient experiences (Henrich et al., 2015). Practitioners should also be mindful to avoid possible stigmatization of IBS patients who may have already developed avoidance behaviors.

In a study comparing the perceptions of patients and gastroenterologists, Levy et al. (2014) found that physicians do not view IBS as a chronic disease to the same extent as other, non-functional gastrointestinal disorders. The authors conclude that this could be due to IBS being viewed as a ‘nondisease’, which in turn can lead to patients’ symptoms not being taken seriously. A previous study investigating patient perspectives on IBS also found that interactions with HCPs did not always contribute to a better understanding of IBS or its treatment (Bertram et al., 2001). Seeking treatment and interacting with HCPs could thereby have an impact on illness perceptions and coping responses. Future research should also investigate the perceptions of HCPs, patient experiences of interactions and their potential effects on illness perceptions, coping behavior and quality of life in IBS.

When interpreting and generalizing from these findings, several limitations should be considered. First, given the cross-sectional nature of the study, no conclusions regarding the temporal links between the different variables can be made. In order to better understand the relationships between illness perceptions, avoidance behaviors, and quality of life in IBS, future studies should aim to use longitudinal methods. Second, as participants were volunteers recruited through social media and word of mouth, the study sample may have suffered from self-selection bias. This could limit the generalizability of the finding to clinical settings. Third, this study did not investigate comorbidity, symptom severity, GI-specific anxiety or symptom catastrophizing, which have been shown to be related to illness perceptions, coping strategies and quality of life (Knowles et al., 2017; Ng & Chow, 2012; Torkzadeh et al., 2019;Trindade et al., 2022). Fourth, this study used the ROME III criteria, which has since then been replaced with a more specific ROME IV criteria. While the potential impact of this is unclear, patients with less severe symptoms might not have filled the newer ROME IV criteria (Aziz et al., 2018). This study was also conducted solely in the Netherlands, which means that the sample was not necessarily culturally diverse and limited to a specific healthcare context. Studies have found differences in not only the prevalence of IBS, but also the quality of life of IBS patients in different countries (Hahn et al., 1999; Lovell & Ford, 2012). This would suggest that cultural and socioeconomic factors might be important.

Finally, it is also important to note that treatment-seeking was self-reported and based only on whether or not the respondent reported receiving an IBS diagnosis from a doctor. This does not give an in-depth overview of treatment-seeking behavior, as it lacks details about frequency of healthcare use, type of treatment sought, medication use, what kind of HCPs respondents had interacted with, performed examinations or referrals received (e.g. to psychiatrist or dietitian). Gathering this data through clinical records and more detailed questions, as done in some previous studies (Ringström et al., 2007; Schwille-Kiuntke et al., 2021), would have been beneficial in order to better understand treatment-seeking behavior. Answering the research questions of this study would however have been impossible with clinical data alone, as data from non-patients was also needed.

In conclusion, illness perceptions and avoidant coping strategies are associated with quality of life in people with IBS symptoms. More specifically, perceiving IBS as a permanent condition with more severe emotional, social and practical consequences predicts more frequent use of avoidant coping strategies, which in turn predicts lower quality of life. These seemingly causal chains, from illness perceptions through avoidant coping strategies and on to quality of life, are significantly more pronounced among people who report seeking treatment for their IBS symptoms. Further research is needed to identify ways in which people with IBS symptoms might be aided to prevent the development of suboptimal illness perceptions and avoidant coping strategies, and thereby maintain their quality of life.

## Supplementary Material

Supplemental Material

## Data Availability

All data and syntax are available from the project’s Open Science Framework page (https://osf.io/cwkfq)
