# Supplementary material for: Examining interactions of illness perceptions, avoidance behavior and patient status in predicting quality of life among people with irritable bowel syndrome
Source: Health Psychol Behav Med. 2024 Feb 5;12(1):2311986. doi: 10.1080/21642850.2024.2311986 (PMC10846424; doi:10.1080/21642850.2024.2311986)
Supplement: Supplemental Material [file RHPB_A_2311986_SM9324.pdf]

Examining interactions of illness perceptions, avoidance behavior and patient status in predicting quality of life among people with irritable bowel syndrome

<u>Supplementary material</u>

Supplement I: Analysis output for moderated mediation model, including all variables and output of simplified moderated mediation model without ROME III filter

Supplement II: Analysis output for moderated mediation model, including all variables and output of simplified moderated mediation model withROME III filter

Supplement III: t-tests, ANOVAs, and correlations for demographic variables and IBS-QoL

Supplement I: Analysis output for moderated mediation model, including all variables and output of simplified moderated mediation model without ROME III filter

Results

GLM Mediation Analysis

Mediators Models		
	m1	UCL_Depressive ~ timeline + timecycl + conseque + perscon + treatcon + illcoher + emotrepr + treatment_seeking + timeline:treatment_seeking + timecycl:treatment_seeking + conseque:treatment_seeking + perscon:treatment_seeking + treatcon:treatment_seeking + illcoher:treatment_seeking + emotrepr:treatment_seeking
	m2	UCL_Palliative ~ timeline + timecycl + conseque + perscon + treatcon + illcoher + emotrepr + treatment_seeking + timeline:treatment_seeking + timecycl:treatment_seeking + conseque:treatment_seeking + perscon:treatment_seeking + treatcon:treatment_seeking + illcoher:treatment_seeking + emotrepr:treatment_seeking
	m3	UCL_Avoidant ~ timeline + timecycl + conseque + perscon + treatcon + illcoher + emotrepr + treatment_seeking + timeline:treatment_seeking + timecycl:treatment_seeking + conseque:treatment_seeking + perscon:treatment_seeking + treatcon:treatment_seeking + illcoher:treatment_seeking + emotrepr:treatment_seeking
	m4	AvoidanceBehavior ~ timeline + timecycl + conseque + perscon + treatcon + illcoher + emotrepr + treatment_seeking + timeline:treatment_seeking + timecycl:treatment_seeking + conseque:treatment_seeking + perscon:treatment_seeking + treatcon:treatment_seeking + illcoher:treatment_seeking + emotrepr:treatment_seeking
Full Model		
	m5	IBSQoL ~ UCL_Depressive + UCL_Palliative + UCL_Avoidant + AvoidanceBehavior + timeline + timecycl + conseque + perscon + treatcon + illcoher + emotrepr + treatment_seeking + UCL_Depressive:treatment_seeking + UCL_Palliative:treatment_seeking + UCL_Avoidant:treatment_seeking + AvoidanceBehavior:treatment_seeking + timeline:treatment_seeking + timecycl:treatment_seeking + conseque:treatment_seeking + perscon:treatment_seeking + treatcon:treatment_seeking + illcoher:treatment_seeking + emotrepr:treatment_seeking
Indirect Effects		
	IE 1	timeline ⇒ UCL_Depressive ⇒ IBSQoL
	IE 2	timeline ⇒ UCL_Palliative ⇒ IBSQoL
	IE 3	timeline ⇒ UCL_Avoidant ⇒ IBSQoL
	IE 4	timeline ⇒ AvoidanceBehavior ⇒ IBSQoL
	IE 5	timecycl ⇒ UCL_Depressive ⇒ IBSQoL
	IE 6	timecycl ⇒ UCL_Palliative ⇒ IBSQoL
	IE 7	timecycl ⇒ UCL_Avoidant ⇒ IBSQoL
	IE 8	timecycl ⇒ AvoidanceBehavior ⇒ IBSQoL
	IE 9	conseque ⇒ UCL_Depressive ⇒ IBSQoL
	IE 10	conseque ⇒ UCL_Palliative ⇒ IBSQoL
	IE 11	conseque ⇒ UCL_Avoidant ⇒ IBSQoL
	IE 12	conseque ⇒ AvoidanceBehavior ⇒ IBSQoL
	IE 13	perscon ⇒ UCL_Depressive ⇒ IBSQoL
	IE 14	perscon ⇒ UCL_Palliative ⇒ IBSQoL
	IE 15	perscon ⇒ UCL_Avoidant ⇒ IBSQoL
	IE 16	perscon ⇒ AvoidanceBehavior ⇒ IBSQoL

[3]

IE 17	treatcon ⇒ UCL_Depressive ⇒ IBSQoL
IE 18	treatcon ⇒ UCL_Palliative ⇒ IBSQoL
IE 19	treatcon ⇒ UCL_Avoidant ⇒ IBSQoL
IE 20	treatcon ⇒ AvoidanceBehavior ⇒ IBSQoL
IE 21	illcoher ⇒ UCL_Depressive ⇒ IBSQoL
IE 22	illcoher ⇒ UCL_Palliative ⇒ IBSQoL
IE 23	illcoher ⇒ UCL_Avoidant ⇒ IBSQoL
IE 24	illcoher ⇒ AvoidanceBehavior ⇒ IBSQoL
IE 25	emotrepr ⇒ UCL_Depressive ⇒ IBSQoL
IE 26	emotrepr ⇒ UCL_Palliative ⇒ IBSQoL
IE 27	emotrepr ⇒ UCL_Avoidant ⇒ IBSQoL
IE 28	emotrepr ⇒ AvoidanceBehavior ⇒ IBSQoL

[3]

Path Model

Model Diagram

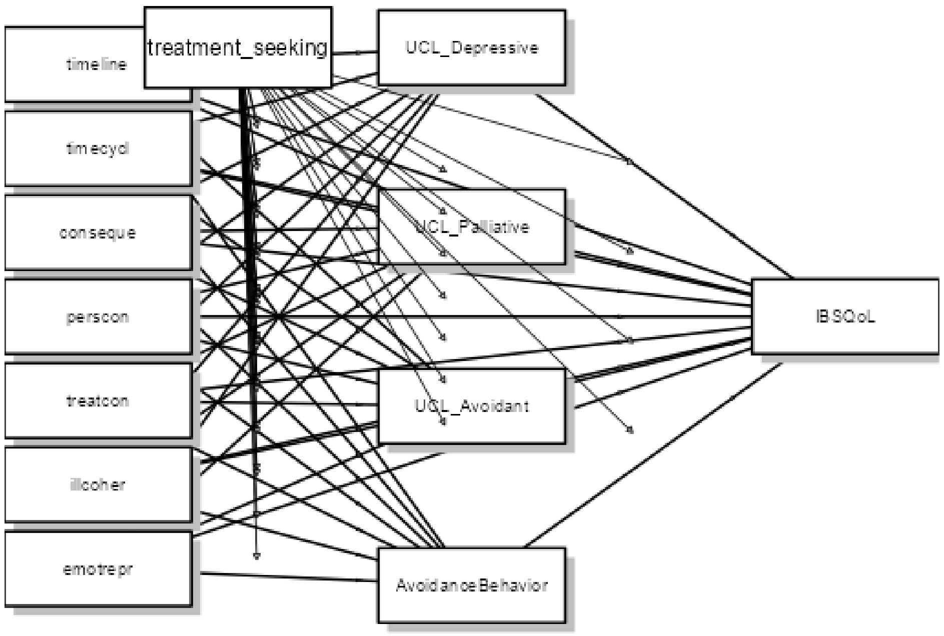

[4]

Model diagram notes
Moderators main effects are not shown
Covariances among IV are estimated but not shown

Mediation

Moderation effects (interactions)

Moderator	Interaction	Estimate	SE	Lower	Upper	β	z	p
	timeline:treatment_seeking1 ⇒ UCL_Depressive	0.06045	0.1203	-0.1754	0.2963	0.03841	0.5024	0.615
	timecycl:treatment_seeking1 ⇒ UCL_Depressive	0.01435	0.1639	-0.3069	0.3356	0.00695	0.0875	0.930
	conseque:treatment_seeking1 ⇒ UCL_Depressive	-0.04348	0.1298	-0.2979	0.2110	-0.03295	-0.3349	0.738
	perscon:treatment_seeking1 ⇒ UCL_Depressive	-0.06975	0.1258	-0.3163	0.1768	-0.03963	-0.5544	0.579
	treatcon:treatment_seeking1 ⇒ UCL_Depressive	-0.33586	0.1588	-0.6472	-0.0246	-0.17275	-2.1145	0.034
	illcoher:treatment_seeking1 ⇒ UCL_Depressive	-0.21577	0.1257	-0.4621	0.0306	-0.13810	-1.7166	0.086
	emotrepr:treatment_seeking1 ⇒ UCL_Depressive	0.03648	0.1335	-0.2252	0.2981	0.02786	0.2732	0.785
	timeline:treatment_seeking1 ⇒ UCL_Palliative	-0.07924	0.1426	-0.3587	0.2002	-0.04897	-0.5558	0.578
	timecycl:treatment_seeking1 ⇒ UCL_Palliative	-0.24850	0.1942	-0.6292	0.1322	-0.11700	-1.2793	0.201
	conseque:treatment_seeking1 ⇒ UCL_Palliative	0.02327	0.1538	-0.2783	0.3248	0.01715	0.1512	0.880
	perscon:treatment_seeking1 ⇒ UCL_Palliative	-0.00526	0.1491	-0.2974	0.2869	-0.00291	-0.0353	0.972
	treatcon:treatment_seeking1 ⇒ UCL_Palliative	0.03530	0.1882	-0.3336	0.4042	0.01766	0.1876	0.851
	illcoher:treatment_seeking1 ⇒ UCL_Palliative	-0.26121	0.1489	-0.5531	0.0307	-0.16258	-1.7537	0.079
	emotrepr:treatment_seeking1 ⇒ UCL_Palliative	0.09223	0.1582	-0.2178	0.4023	0.06850	0.5830	0.560
	timeline:treatment_seeking1 ⇒ UCL_Avoidant	-0.13971	0.1573	-0.4480	0.1686	-0.07616	-0.8882	0.374
	timecycl:treatment_seeking1 ⇒ UCL_Avoidant	0.15135	0.2143	-0.2687	0.5714	0.06286	0.7062	0.480
	conseque:treatment_seeking1 ⇒ UCL_Avoidant	0.20328	0.1697	-0.1294	0.5360	0.13217	1.1976	0.231
	perscon:treatment_seeking1 ⇒ UCL_Avoidant	0.20185	0.1645	-0.1205	0.5242	0.09839	1.2273	0.220
	treatcon:treatment_seeking1 ⇒ UCL_Avoidant	-0.23488	0.2077	-0.6419	0.1721	-0.10363	-1.1311	0.258
	illcoher:treatment_seeking1 ⇒ UCL_Avoidant	-0.26201	0.1643	-0.5841	0.0601	-0.14385	-1.5944	0.111
	emotrepr:treatment_seeking1 ⇒ UCL_Avoidant	0.00499	0.1745	-0.3371	0.3471	0.00327	0.0286	0.977
	timeline:treatment_seeking1 ⇒ AvoidanceBehavior	0.95658	0.7529	-0.5191	2.4322	0.08420	1.2705	0.204
	timecycl:treatment_seeking1 ⇒ AvoidanceBehavior	-0.85088	1.0258	-2.8614	1.1597	-0.05706	-0.8295	0.407
	conseque:treatment_seeking1 ⇒ AvoidanceBehavior	-0.21252	0.8125	-1.8050	1.3799	-0.02231	-0.2616	0.794
	perscon:treatment_seeking1 ⇒ AvoidanceBehavior	0.34505	0.7872	-1.1979	1.8880	0.02716	0.4383	0.661
	treatcon:treatment_seeking1 ⇒ AvoidanceBehavior	0.25897	0.9940	-1.6892	2.2071	0.01845	0.2605	0.794
	illcoher:treatment_seeking1 ⇒ AvoidanceBehavior	-0.75763	0.7866	-2.2993	0.7841	-0.06716	-0.9632	0.335
	emotrepr:treatment_seeking1 ⇒ AvoidanceBehavior	0.97702	0.8355	-0.6604	2.6145	0.10335	1.1694	0.242
	UCL_Depressive:treatment_seeking1 ⇒ IBSQoL	-0.08845	0.5601	-1.1862	1.0093	-0.02385	-0.1579	0.875
	UCL_Palliative:treatment_seeking1 ⇒ IBSQoL	0.07414	0.4543	-0.8162	0.9645	0.02378	0.1632	0.870
	UCL_Avoidant:treatment_seeking1 ⇒ IBSQoL	0.53240	0.4361	-0.3223	1.3871	0.21125	1.2209	0.222
	AvoidanceBehavior:treatment_seeking1 ⇒ IBSQoL	0.05562	0.0855	-0.1119	0.2231	0.05080	0.6509	0.515

Moderation effects (interactions)

Moderator	Interaction	Estimate	SE	Lower	Upper	β	z	p
	timeline:treatment_seeking1 ⇒ IBSQoL	-0.31883	0.4905	-1.2802	0.6426	-0.03372	-0.6500	0.516
	timecycl:treatment_seeking1 ⇒ IBSQoL	1.29753	0.6535	0.0167	2.5783	0.10455	1.9855	0.047
	conseque:treatment_seeking1 ⇒ IBSQoL	1.15484	0.5532	0.0707	2.2390	0.14566	2.0877	0.037
	perscon:treatment_seeking1 ⇒ IBSQoL	0.09813	0.4998	-0.8815	1.0777	0.00928	0.1963	0.844
	treatcon:treatment_seeking1 ⇒ IBSQoL	-0.26771	0.6436	-1.5291	0.9937	-0.02292	-0.4160	0.677
	illcoher:treatment_seeking1 ⇒ IBSQoL	0.46871	0.5107	-0.5323	1.4697	0.04992	0.9177	0.359
	emotrepr:treatment_seeking1 ⇒ IBSQoL	-0.65873	0.5431	-1.7231	0.4057	-0.08372	-1.2130	0.225

Conditional Mediation

Moderator levels					95% C.I. (a)				
treatment_seeking	Type	Effect	Estimate	SE	Lower	Upper	β	z	p
Average	Indirect	timeline ⇒ UCL_Depressive ⇒ IBSQoL	-0.01304	0.05691	-0.12459	0.0985	-0.00284	-0.2291	0.819
Average		timeline ⇒ UCL_Palliative ⇒ IBSQoL	0.03243	0.04129	-0.04849	0.1134	0.00707	0.7855	0.432
Average		timeline ⇒ UCL_Avoidant ⇒ IBSQoL	-0.02885	0.03628	-0.09995	0.0423	-0.00629	-0.7951	0.427
Average		timeline ⇒ AvoidanceBehavior ⇒ IBSQoL	0.25604	0.13121	-0.00112	0.5132	0.05579	1.9514	0.051
Average		timecycl ⇒ UCL_Depressive ⇒ IBSQoL	0.00255	0.07738	-0.14912	0.1542	4.12e-4	0.0330	0.974
Average		timecycl ⇒ UCL_Palliative ⇒ IBSQoL	0.02759	0.03784	-0.04657	0.1018	0.00446	0.7293	0.466
Average		timecycl ⇒ UCL_Avoidant ⇒ IBSQoL	-0.00924	0.02153	-0.05144	0.0330	-0.00149	-0.4293	0.668
Average		timecycl ⇒ AvoidanceBehavior ⇒ IBSQoL	0.08788	0.17370	-0.25256	0.4283	0.01420	0.5059	0.613
Average		conseque ⇒ UCL_Depressive ⇒ IBSQoL	0.12874	0.07114	-0.01070	0.2682	0.03363	1.8095	0.070
Average		conseque ⇒ UCL_Palliative ⇒ IBSQoL	-0.02124	0.02932	-0.07870	0.0362	-0.00555	-0.7244	0.469
Average		conseque ⇒ UCL_Avoidant ⇒ IBSQoL	-0.01086	0.01944	-0.04896	0.0272	-0.00284	-0.5584	0.577
Average		conseque ⇒ AvoidanceBehavior ⇒ IBSQoL	0.75591	0.16679	0.42901	1.0828	0.19747	4.5322	< .001
Average		perscon ⇒ UCL_Depressive ⇒ IBSQoL	0.02451	0.05978	-0.09266	0.1417	0.00464	0.4099	0.682
Average		perscon ⇒ UCL_Palliative ⇒ IBSQoL	-0.00253	0.01415	-0.03026	0.0252	-4.79e−4	-0.1788	0.858
Average		perscon ⇒ UCL_Avoidant ⇒ IBSQoL	0.01055	0.01886	-0.02641	0.0475	0.00200	0.5593	0.576
Average		perscon ⇒ AvoidanceBehavior ⇒ IBSQoL	-0.12963	0.13402	-0.39231	0.1330	-0.02456	-0.9672	0.333
Average		treatcon ⇒ UCL_Depressive ⇒ IBSQoL	0.08853	0.07899	-0.06628	0.2433	0.01529	1.1208	0.262
Average		treatcon ⇒ UCL_Palliative ⇒ IBSQoL	0.02842	0.03846	-0.04696	0.1038	0.00491	0.7389	0.460
Average		treatcon ⇒ UCL_Avoidant ⇒ IBSQoL	-0.01832	0.02796	-0.07312	0.0365	-0.00316	-0.6553	0.512

Note. Confidence intervals computed with method: Standard (Delta method)

Note. Betas are completely standardized effect sizes

[5]

Conditional Mediation

Moderator levels					95% C.I. (a)				
treatment_seeking	Type	Effect	Estimate	SE	Lower	Upper	β	z	p
Average		treatcon ⇒ AvoidanceBehavior ⇒ IBSQoL	-0.24938	0.17085	-0.58424	0.0855	-0.04307	-1.4597	0.144
Average		illcoher ⇒ UCL_Depressive ⇒ IBSQoL	0.00846	0.05938	-0.10793	0.1249	0.00181	0.1425	0.887
Average		illcoher ⇒ UCL_Palliative ⇒ IBSQoL	0.02398	0.03205	-0.03883	0.0868	0.00513	0.7483	0.454
Average		illcoher ⇒ UCL_Avoidant ⇒ IBSQoL	-0.01168	0.01974	-0.05036	0.0270	-0.00250	-0.5919	0.554
Average		illcoher ⇒ AvoidanceBehavior ⇒ IBSQoL	0.17337	0.13469	-0.09062	0.4373	0.03706	1.2872	0.198
Average		emotrepr ⇒ UCL_Depressive ⇒ IBSQoL	0.20634	0.08558	0.03860	0.3741	0.05330	2.4110	0.016
Average		emotrepr ⇒ UCL_Palliative ⇒ IBSQoL	0.01438	0.02271	-0.03013	0.0589	0.00371	0.6331	0.527
Average		emotrepr ⇒ UCL_Avoidant ⇒ IBSQoL	-0.01591	0.02396	-0.06286	0.0310	-0.00411	-0.6641	0.507
Average		emotrepr ⇒ AvoidanceBehavior ⇒ IBSQoL	0.20609	0.14352	-0.07520	0.4874	0.05324	1.4360	0.151
Average	Component	timeline ⇒ UCL_Depressive	-0.01381	0.06016	-0.13171	0.1041	-0.01808	-0.2296	0.818
Average		UCL_Depressive ⇒ IBSQoL	0.94412	0.26494	0.42485	1.4634	0.15712	3.5635	< .001
Average		timeline ⇒ UCL_Palliative	0.17496	0.07128	0.03525	0.3147	0.22275	2.4544	0.014
Average		UCL_Palliative ⇒ IBSQoL	0.18538	0.22358	-0.25284	0.6236	0.03172	0.8291	0.407
Average		timeline ⇒ UCL_Avoidant	0.16581	0.07865	0.01166	0.3200	0.18621	2.1083	0.035
Average		UCL_Avoidant ⇒ IBSQoL	-0.17398	0.20265	-0.57116	0.2232	-0.03375	-0.8586	0.391
Average		timeline ⇒ AvoidanceBehavior	0.75759	0.37645	0.01976	1.4954	0.13738	2.0125	0.044
Average		AvoidanceBehavior ⇒ IBSQoL	0.33797	0.04234	0.25500	0.4210	0.40608	7.9830	< .001
Average		timecycl ⇒ UCL_Depressive	0.00270	0.08196	-0.15794	0.1633	0.00262	0.0330	0.974
Average		timecycl ⇒ UCL_Palliative	0.14886	0.09712	-0.04150	0.3392	0.14058	1.5327	0.125
Average		timecycl ⇒ UCL_Avoidant	0.05313	0.10715	-0.15689	0.2631	0.04426	0.4958	0.620
Average		timecycl ⇒ AvoidanceBehavior	0.26001	0.51290	-0.74526	1.2653	0.03497	0.5069	0.612
Average		conseque ⇒ UCL_Depressive	0.13636	0.06492	0.00912	0.2636	0.21405	2.1005	0.036
Average		conseque ⇒ UCL_Palliative	-0.11457	0.07692	-0.26534	0.0362	-0.17490	-1.4894	0.136

Note. Confidence intervals computed with method: Standard (Delta method)

Note. Betas are completely standardized effect sizes

[5]

Conditional Mediation

Moderator levels					95% C.I. (a)				
treatment_seeking	Type	Effect	Estimate	SE	Lower	Upper	β	z	p
Average		conseque ⇒ UCL_Avoidant	0.06239	0.08487	-0.10395	0.2287	0.08401	0.7351	0.462
Average		conseque ⇒ AvoidanceBehavior	2.23658	0.40625	1.44035	3.0328	0.48628	5.5055	< .001
Average		perscon ⇒ UCL_Depressive	0.02596	0.06290	-0.09732	0.1492	0.02956	0.4127	0.680
Average		perscon ⇒ UCL_Palliative	-0.01365	0.07453	-0.15973	0.1324	-0.01511	-0.1831	0.855
Average		perscon ⇒ UCL_Avoidant	-0.06062	0.08223	-0.22180	0.1006	-0.05921	-0.7371	0.461
Average		perscon ⇒ AvoidanceBehavior	-0.38356	0.39362	-1.15504	0.3879	-0.06049	-0.9744	0.330
Average		treatcon ⇒ UCL_Depressive	0.09377	0.07942	-0.06189	0.2494	0.09730	1.1807	0.238
Average		treatcon ⇒ UCL_Palliative	0.15330	0.09411	-0.03114	0.3377	0.15470	1.6290	0.103
Average		treatcon ⇒ UCL_Avoidant	0.10529	0.10383	-0.09821	0.3088	0.09373	1.0141	0.311
Average		treatcon ⇒ AvoidanceBehavior	-0.73787	0.49698	-1.71194	0.2362	-0.10605	-1.4847	0.138
Average		illcoher ⇒ UCL_Depressive	0.00896	0.06285	-0.11422	0.1321	0.01151	0.1426	0.887
Average		illcoher ⇒ UCL_Palliative	0.12936	0.07447	-0.01660	0.2753	0.16159	1.7370	0.082
Average		illcoher ⇒ UCL_Avoidant	0.06714	0.08217	-0.09391	0.2282	0.07397	0.8171	0.414
Average		illcoher ⇒ AvoidanceBehavior	0.51296	0.39330	-0.25790	1.2838	0.09126	1.3042	0.192
Average		emotrepr ⇒ UCL_Depressive	0.21855	0.06675	0.08772	0.3494	0.33924	3.2741	0.001
Average		emotrepr ⇒ UCL_Palliative	0.07755	0.07910	-0.07748	0.2326	0.11706	0.9804	0.327
Average		emotrepr ⇒ UCL_Avoidant	0.09144	0.08727	-0.07960	0.2625	0.12176	1.0478	0.295
Average		emotrepr ⇒ AvoidanceBehavior	0.60979	0.41773	-0.20894	1.4285	0.13110	1.4598	0.144
Average	Direct	timeline ⇒ IBSQoL	0.68560	0.24641	0.20265	1.1685	0.14938	2.7824	0.005
Average		timecycl ⇒ IBSQoL	-0.75141	0.32730	-1.39291	-0.1099	-0.12144	-2.2958	0.022
Average		conseque ⇒ IBSQoL	-0.01487	0.27766	-0.55908	0.5293	-0.00388	-0.0536	0.957
Average		perscon ⇒ IBSQoL	-0.09359	0.25019	-0.58396	0.3968	-0.01774	-0.3741	0.708
Average		treatcon ⇒ IBSQoL	0.14846	0.32139	-0.48146	0.7784	0.02564	0.4619	0.644
Average		illcoher ⇒ IBSQoL	-0.38390	0.25442	-0.88255	0.1147	-0.08206	-1.5089	0.131
Average		emotrepr ⇒ IBSQoL	1.35533	0.27264	0.82096	1.8897	0.35011	4.9711	< .001
Average	Total	timeline ⇒ IBSQoL	0.92406	0.28195	0.37144	1.4767	0.19996	3.2774	0.001
Average		timecycl ⇒ IBSQoL	-0.63926	0.38415	-1.39219	0.1137	-0.10261	-1.6641	0.096
Average		conseque ⇒ IBSQoL	0.86318	0.30427	0.26682	1.4595	0.22396	2.8369	0.005
Average		perscon ⇒ IBSQoL	-0.15758	0.29482	-0.73541	0.4202	-0.02966	-0.5345	0.593
Average		treatcon ⇒ IBSQoL	-0.02194	0.37223	-0.75150	0.7076	-0.00376	-0.0589	0.953
Average		illcoher ⇒ IBSQoL	-0.23524	0.29457	-0.81259	0.3421	-0.04994	-0.7986	0.425
Average		emotrepr ⇒ IBSQoL	1.78139	0.31287	1.16818	2.3946	0.45705	5.6937	< .001
no	Indirect	timeline ⇒ UCL_Depressive ⇒ IBSQoL	-0.04354	0.10335	-0.24610	0.1590	-0.00949	-0.4213	0.674

Note. Confidence intervals computed with method: Standard (Delta method)

Note. Betas are completely standardized effect sizes

[5]

Conditional Mediation

Moderator levels					95% C.I. (a)				
treatment_seeking	Type	Effect	Estimate	SE	Lower	Upper	β	z	p
no		timeline ⇒ UCL_Palliative ⇒ IBSQoL	0.03177	0.05132	-0.06882	0.1324	0.00692	0.6190	0.536
no		timeline ⇒ UCL_Avoidant ⇒ IBSQoL	-0.10373	0.07652	-0.25370	0.0462	-0.02260	-1.3556	0.175
no		timeline ⇒ AvoidanceBehavior ⇒ IBSQoL	0.08662	0.20202	-0.30933	0.4826	0.01887	0.4288	0.668
no		timecycl ⇒ UCL_Depressive ⇒ IBSQoL	-0.00443	0.14731	-0.29316	0.2843	-7.16e−4	-0.0301	0.976
no		timecycl ⇒ UCL_Palliative ⇒ IBSQoL	0.04043	0.06642	-0.08976	0.1706	0.00653	0.6087	0.543
no		timecycl ⇒ UCL_Avoidant ⇒ IBSQoL	0.00993	0.08588	-0.15840	0.1783	0.00160	0.1156	0.908
no		timecycl ⇒ AvoidanceBehavior ⇒ IBSQoL	0.21253	0.29076	-0.35735	0.7824	0.03434	0.7309	0.465
no		conseque ⇒ UCL_Depressive ⇒ IBSQoL	0.15627	0.12321	-0.08522	0.3978	0.04082	1.2683	0.205
no		conseque ⇒ UCL_Palliative ⇒ IBSQoL	-0.01868	0.03492	-0.08712	0.0498	-0.00488	-0.5351	0.593
no		conseque ⇒ UCL_Avoidant ⇒ IBSQoL	0.01728	0.06793	-0.11585	0.1504	0.00451	0.2544	0.799
no		conseque ⇒ AvoidanceBehavior ⇒ IBSQoL	0.72682	0.24825	0.24026	1.2134	0.18985	2.9278	0.003
no		perscon ⇒ UCL_Depressive ⇒ IBSQoL	0.06013	0.10716	-0.14989	0.2702	0.01139	0.5612	0.575
no		perscon ⇒ UCL_Palliative ⇒ IBSQoL	-0.00163	0.01896	-0.03880	0.0355	-3.09e−4	-0.0860	0.931
no		perscon ⇒ UCL_Avoidant ⇒ IBSQoL	0.07110	0.06983	-0.06575	0.2080	0.01347	1.0183	0.309
no		perscon ⇒ AvoidanceBehavior ⇒ IBSQoL	-0.17249	0.20939	-0.58288	0.2379	-0.03268	-0.8238	0.410
no		treatcon ⇒ UCL_Depressive ⇒ IBSQoL	0.25864	0.15725	-0.04958	0.5668	0.04466	1.6447	0.100
no		treatcon ⇒ UCL_Palliative ⇒ IBSQoL	0.02008	0.03934	-0.05702	0.0972	0.00347	0.5105	0.610
no		treatcon ⇒ UCL_Avoidant ⇒ IBSQoL	-0.09804	0.09375	-0.28179	0.0857	-0.01693	-1.0457	0.296
no		treatcon ⇒ AvoidanceBehavior ⇒ IBSQoL	-0.26906	0.27963	-0.81713	0.2790	-0.04646	-0.9622	0.336

Note. Confidence intervals computed with method: Standard (Delta method)

Note. Betas are completely standardized effect sizes

[5]

Conditional Mediation

Moderator levels					95% C.I. (a)				
treatment_seeking	Type	Effect	Estimate	SE	Lower	Upper	β	z	p
no		illcoher ⇒ UCL_Depressive ⇒ IBSQoL	0.11550	0.11297	-0.10592	0.3369	0.02469	1.0224	0.307
no		illcoher ⇒ UCL_Palliative ⇒ IBSQoL	0.03849	0.06124	-0.08154	0.1585	0.00823	0.6285	0.530
no		illcoher ⇒ UCL_Avoidant ⇒ IBSQoL	-0.08721	0.07492	-0.23405	0.0596	-0.01864	-1.1641	0.244
no		illcoher ⇒ AvoidanceBehavior ⇒ IBSQoL	0.27659	0.21669	-0.14811	0.7013	0.05912	1.2764	0.202
no		emotrepr ⇒ UCL_Depressive ⇒ IBSQoL	0.19800	0.13214	-0.06099	0.4570	0.05114	1.4984	0.134
no		emotrepr ⇒ UCL_Palliative ⇒ IBSQoL	0.00465	0.02260	-0.03964	0.0489	0.00120	0.2059	0.837
no		emotrepr ⇒ UCL_Avoidant ⇒ IBSQoL	-0.03915	0.07272	-0.18168	0.1034	-0.01011	-0.5384	0.590
no		emotrepr ⇒ AvoidanceBehavior ⇒ IBSQoL	0.03756	0.23772	-0.42838	0.5035	0.00970	0.1580	0.874
no	Component	timeline ⇒ UCL_Depressive	-0.04405	0.10389	-0.24767	0.1596	-0.05767	-0.4240	0.672
no		UCL_Depressive ⇒ IBSQoL	0.98843	0.26494	0.46916	1.5077	0.16448	3.7308	< .001
no		timeline ⇒ UCL_Palliative	0.21457	0.12311	-0.02671	0.4559	0.27319	1.7430	0.081
no		UCL_Palliative ⇒ IBSQoL	0.14805	0.22358	-0.29017	0.5863	0.02533	0.6622	0.508
no		timeline ⇒ UCL_Avoidant	0.23567	0.13582	-0.03055	0.5019	0.26467	1.7351	0.083
no		UCL_Avoidant ⇒ IBSQoL	-0.44015	0.20265	-0.83733	-0.0430	-0.08538	-2.1720	0.030
no		timeline ⇒ AvoidanceBehavior	0.27925	0.65014	-0.99500	1.5535	0.05064	0.4295	0.668
no		AvoidanceBehavior ⇒ IBSQoL	0.31020	0.04234	0.22723	0.3932	0.37268	7.3271	< .001
no		timecycl ⇒ UCL_Depressive	-0.00448	0.14903	-0.29658	0.2876	-0.00435	-0.0301	0.976
no		timecycl ⇒ UCL_Palliative	0.27310	0.17660	-0.07303	0.6192	0.25792	1.5465	0.122
no		timecycl ⇒ UCL_Avoidant	-0.02255	0.19485	-0.40444	0.3593	-0.01879	-0.1158	0.908
no		timecycl ⇒ AvoidanceBehavior	0.68513	0.93265	-1.14282	2.5131	0.09215	0.7346	0.463
no		conseque ⇒ UCL_Depressive	0.15810	0.11723	-0.07166	0.3879	0.24818	1.3487	0.177
no		conseque ⇒ UCL_Palliative	-0.12621	0.13891	-0.39847	0.1461	-0.19266	-0.9085	0.364
no		conseque ⇒ UCL_Avoidant	-0.03925	0.15326	-0.33964	0.2611	-0.05286	-0.2561	0.798
no		conseque ⇒ AvoidanceBehavior	2.34304	0.73361	0.90519	3.7809	0.50943	3.1938	0.001

Note. Confidence intervals computed with method: Standard (Delta method)

Note. Betas are completely standardized effect sizes

[5]

Conditional Mediation

Moderator levels					95% C.I. (a)				
treatment_seeking	Type	Effect	Estimate	SE	Lower	Upper	β	z	p
no		perscon ⇒ UCL_Depressive	0.06084	0.10718	-0.14923	0.2709	0.06927	0.5676	0.570
no		perscon ⇒ UCL_Palliative	-0.01102	0.12700	-0.25994	0.2379	-0.01220	-0.0868	0.931
no		perscon ⇒ UCL_Avoidant	-0.16155	0.14013	-0.43619	0.1131	-0.15780	-1.1529	0.249
no		perscon ⇒ AvoidanceBehavior	-0.55605	0.67072	-1.87064	0.7585	-0.08770	-0.8290	0.407
no		treatcon ⇒ UCL_Depressive	0.26166	0.14280	-0.01822	0.5415	0.27152	1.8324	0.067
no		treatcon ⇒ UCL_Palliative	0.13565	0.16921	-0.19601	0.4673	0.13689	0.8016	0.423
no		treatcon ⇒ UCL_Avoidant	0.22273	0.18670	-0.14318	0.5887	0.19826	1.1930	0.233
no		treatcon ⇒ AvoidanceBehavior	-0.86736	0.89364	-2.61886	0.8841	-0.12466	-0.9706	0.332
no		illcoher ⇒ UCL_Depressive	0.11685	0.10992	-0.09858	0.3323	0.15009	1.0631	0.288
no		illcoher ⇒ UCL_Palliative	0.25997	0.13025	0.00469	0.5152	0.32473	1.9960	0.046
no		illcoher ⇒ UCL_Avoidant	0.19815	0.14370	-0.08351	0.4798	0.21832	1.3789	0.168
no		illcoher ⇒ AvoidanceBehavior	0.89163	0.68785	-0.45653	2.2398	0.15863	1.2963	0.195
no		emotrepr ⇒ UCL_Depressive	0.20032	0.12243	-0.03964	0.4403	0.31094	1.6362	0.102
no		emotrepr ⇒ UCL_Palliative	0.03144	0.14508	-0.25291	0.3158	0.04745	0.2167	0.828
no		emotrepr ⇒ UCL_Avoidant	0.08895	0.16007	-0.22477	0.4027	0.11845	0.5557	0.578
no		emotrepr ⇒ AvoidanceBehavior	0.12107	0.76617	-1.38060	1.6227	0.02603	0.1580	0.874
no	Direct	timeline ⇒ IBSQoL	0.84530	0.41508	0.03176	1.6588	0.18415	2.0365	0.042
no		timecycl ⇒ IBSQoL	-1.39988	0.59089	-2.55800	-0.2418	-0.22621	-2.3691	0.018
no		conseque ⇒ IBSQoL	-0.59246	0.47501	-1.52347	0.3385	-0.15476	-1.2473	0.212
no		perscon ⇒ IBSQoL	-0.14260	0.42437	-0.97435	0.6892	-0.02702	-0.3360	0.737
no		treatcon ⇒ IBSQoL	0.28291	0.57048	-0.83521	1.4010	0.04885	0.4959	0.620
no		illcoher ⇒ IBSQoL	-0.61831	0.44135	-1.48335	0.2467	-0.13216	-1.4010	0.161
no		emotrepr ⇒ IBSQoL	1.68452	0.48554	0.73289	2.6362	0.43511	3.4694	< .001
no	Total	timeline ⇒ IBSQoL	0.81609	0.48694	-0.13830	1.7705	0.17660	1.6759	0.094
no		timecycl ⇒ IBSQoL	-1.14164	0.69854	-2.51074	0.2275	-0.18325	-1.6343	0.102
no		conseque ⇒ IBSQoL	0.28921	0.54946	-0.78771	1.3661	0.07504	0.5264	0.599
no		perscon ⇒ IBSQoL	-0.18549	0.50236	-1.17009	0.7991	-0.03491	-0.3692	0.712
no		treatcon ⇒ IBSQoL	0.19379	0.66932	-1.11805	1.5056	0.03324	0.2895	0.772
no		illcoher ⇒ IBSQoL	-0.27480	0.51519	-1.28455	0.7349	-0.05834	-0.5334	0.594
no		emotrepr ⇒ IBSQoL	1.88581	0.57385	0.76109	3.0105	0.48384	3.2863	0.001
yes	Indirect	timeline ⇒ UCL_Depressive ⇒ IBSQoL	0.01477	0.05477	-0.09258	0.1221	0.00320	0.2697	0.787
yes		timeline ⇒ UCL_Palliative ⇒ IBSQoL	0.03011	0.03423	-0.03698	0.0972	0.00652	0.8797	0.379

Note. Confidence intervals computed with method: Standard (Delta method)

Note. Betas are completely standardized effect sizes

[5]

Conditional Mediation

Moderator levels					95% C.I. (a)				
treatment_seeking	Type	Effect	Estimate	SE	Lower	Upper	β	z	p
yes		timeline ⇒ UCL_Avoidant ⇒ IBSQoL	0.00885	0.02077	-0.03187	0.0496	0.00192	0.4259	0.670
yes		timeline ⇒ AvoidanceBehavior ⇒ IBSQoL	0.45208	0.14842	0.16119	0.7430	0.09792	3.0460	0.002
yes		timecycl ⇒ UCL_Depressive ⇒ IBSQoL	0.00889	0.06148	-0.11160	0.1294	0.00143	0.1446	0.885
yes		timecycl ⇒ UCL_Palliative ⇒ IBSQoL	0.00547	0.01881	-0.03140	0.0423	8.79e-4	0.2909	0.771
yes		timecycl ⇒ UCL_Avoidant ⇒ IBSQoL	0.01188	0.02737	-0.04176	0.0655	0.00191	0.4340	0.664
yes		timecycl ⇒ AvoidanceBehavior ⇒ IBSQoL	-0.06051	0.15639	-0.36704	0.2460	-0.00972	-0.3869	0.699
yes		conseque ⇒ UCL_Depressive ⇒ IBSQoL	0.10314	0.05868	-0.01188	0.2182	0.02678	1.7575	0.079
yes		conseque ⇒ UCL_Palliative ⇒ IBSQoL	-0.02290	0.02732	-0.07644	0.0306	-0.00595	-0.8384	0.402
yes		conseque ⇒ UCL_Avoidant ⇒ IBSQoL	0.01513	0.03392	-0.05135	0.0816	0.00393	0.4460	0.656
yes		conseque ⇒ AvoidanceBehavior ⇒ IBSQoL	0.77923	0.15637	0.47275	1.0857	0.20236	4.9833	< .001
yes		perscon ⇒ UCL_Depressive ⇒ IBSQoL	-0.00803	0.05932	-0.12428	0.1082	-0.00151	-0.1353	0.892
yes		perscon ⇒ UCL_Palliative ⇒ IBSQoL	-0.00362	0.01774	-0.03839	0.0311	-6.82e−4	-0.2042	0.838
yes		perscon ⇒ UCL_Avoidant ⇒ IBSQoL	0.00372	0.01139	-0.01861	0.0260	7.00e-4	0.3263	0.744
yes		perscon ⇒ AvoidanceBehavior ⇒ IBSQoL	-0.07721	0.15103	-0.37322	0.2188	-0.01455	-0.5112	0.609
yes		treatcon ⇒ UCL_Depressive ⇒ IBSQoL	-0.06674	0.06559	-0.19529	0.0618	-0.01146	-1.0175	0.309
yes		treatcon ⇒ UCL_Palliative ⇒ IBSQoL	0.03803	0.04239	-0.04506	0.1211	0.00653	0.8971	0.370
yes		treatcon ⇒ UCL_Avoidant ⇒ IBSQoL	-0.00112	0.00874	-0.01825	0.0160	-1.92e−4	-0.1282	0.898
yes		treatcon ⇒ AvoidanceBehavior ⇒ IBSQoL	-0.22252	0.16125	-0.53857	0.0935	-0.03820	-1.3800	0.168
yes		illcoher ⇒ UCL_Depressive ⇒ IBSQoL	-0.08902	0.06081	-0.20820	0.0302	-0.01892	-1.4639	0.143

Note. Confidence intervals computed with method: Standard (Delta method)

Note. Betas are completely standardized effect sizes

[5]

Moderator levels					95% C.I. (a)				
treatment_seeking	Type	Effect	Estimate	SE	Lower	Upper	β	z	p
yes		illcoher ⇒ UCL_Palliative ⇒ IBSQoL	-2.77e−4	0.01608	-0.03179	0.0312	-5.89e−5	-0.0172	0.986
yes		illcoher ⇒ UCL_Avoidant ⇒ IBSQoL	-0.00589	0.01489	-0.03507	0.0233	-0.00125	-0.3957	0.692
yes		illcoher ⇒ AvoidanceBehavior ⇒ IBSQoL	0.04909	0.13969	-0.22470	0.3229	0.01043	0.3514	0.725
yes		emotrepr ⇒ UCL_Depressive ⇒ IBSQoL	0.21309	0.07893	0.05838	0.3678	0.05472	2.6996	0.007
yes		emotrepr ⇒ UCL_Palliative ⇒ IBSQoL	0.02751	0.03101	-0.03326	0.0883	0.00706	0.8873	0.375
yes		emotrepr ⇒ UCL_Avoidant ⇒ IBSQoL	0.00866	0.02009	-0.03071	0.0480	0.00222	0.4312	0.666
yes		emotrepr ⇒ AvoidanceBehavior ⇒ IBSQoL	0.40173	0.13042	0.14612	0.6573	0.10317	3.0804	0.002
yes	Component	timeline ⇒ UCL_Depressive	0.01641	0.06068	-0.10251	0.1353	0.02149	0.2705	0.787
yes		UCL_Depressive ⇒ IBSQoL	0.89988	0.26494	0.38061	1.4192	0.14887	3.3965	< .001
yes		timeline ⇒ UCL_Palliative	0.13534	0.07190	-0.00558	0.2763	0.17232	1.8824	0.060
yes		UCL_Palliative ⇒ IBSQoL	0.22246	0.22358	-0.21576	0.6607	0.03784	0.9950	0.320
yes		timeline ⇒ UCL_Avoidant	0.09595	0.07933	-0.05953	0.2514	0.10776	1.2096	0.226
yes		UCL_Avoidant ⇒ IBSQoL	0.09222	0.20265	-0.30496	0.4894	0.01779	0.4551	0.649
yes		timeline ⇒ AvoidanceBehavior	1.23594	0.37971	0.49173	1.9801	0.22412	3.2550	0.001
yes		AvoidanceBehavior ⇒ IBSQoL	0.36578	0.04234	0.28280	0.4488	0.43690	8.6398	< .001
yes		timecycl ⇒ UCL_Depressive	0.00988	0.06825	-0.12390	0.1437	0.00959	0.1447	0.885
yes		timecycl ⇒ UCL_Palliative	0.02460	0.08088	-0.13392	0.1831	0.02324	0.3042	0.761
yes		timecycl ⇒ UCL_Avoidant	0.12880	0.08924	-0.04609	0.3037	0.10730	1.4434	0.149
yes		timecycl ⇒ AvoidanceBehavior	-0.16543	0.42713	-1.00260	0.6717	-0.02225	-0.3873	0.699
yes		conseque ⇒ UCL_Depressive	0.11461	0.05580	0.00524	0.2240	0.17991	2.0539	0.040
yes		conseque ⇒ UCL_Palliative	-0.10294	0.06613	-0.23255	0.0267	-0.15714	-1.5567	0.120
yes		conseque ⇒ UCL_Avoidant	0.16404	0.07296	0.02104	0.3070	0.22088	2.2484	0.025
yes		conseque ⇒ AvoidanceBehavior	2.13033	0.34922	1.44587	2.8148	0.46318	6.1002	< .001
yes		perscon ⇒ UCL_Depressive	-0.00892	0.06586	-0.13801	0.1202	-0.01016	-0.1354	0.892

Note. Confidence intervals computed with method: Standard (Delta method)

Note. Betas are completely standardized effect sizes

[5]

Conditional Mediation

Moderator levels					95% C.I. (a)				
treatment_seeking	Type	Effect	Estimate	SE	Lower	Upper	β	z	p
yes		perscon ⇒ UCL_Palliative	-0.01628	0.07805	-0.16925	0.1367	-0.01803	-0.2086	0.835
yes		perscon ⇒ UCL_Avoidant	0.04031	0.08611	-0.12846	0.2091	0.03938	0.4682	0.640
yes		perscon ⇒ AvoidanceBehavior	-0.21109	0.41217	-1.01893	0.5968	-0.03329	-0.5121	0.609
yes		treatcon ⇒ UCL_Depressive	-0.07416	0.06954	-0.21046	0.0621	-0.07695	-1.0664	0.286
yes		treatcon ⇒ UCL_Palliative	0.17096	0.08240	0.00945	0.3325	0.17252	2.0747	0.038
yes		treatcon ⇒ UCL_Avoidant	-0.01215	0.09092	-0.19034	0.1660	-0.01081	-0.1336	0.894
yes		treatcon ⇒ AvoidanceBehavior	-0.60835	0.43518	-1.46129	0.2446	-0.08744	-1.3979	0.162
yes		illcoher ⇒ UCL_Depressive	-0.09892	0.06097	-0.21843	0.0206	-0.12706	-1.6223	0.105
yes		illcoher ⇒ UCL_Palliative	-0.00125	0.07225	-0.14286	0.1404	-0.00156	-0.0173	0.986
yes		illcoher ⇒ UCL_Avoidant	-0.06387	0.07972	-0.22012	0.0924	-0.07037	-0.8012	0.423
yes		illcoher ⇒ AvoidanceBehavior	0.13420	0.38158	-0.61368	0.8821	0.02387	0.3517	0.725
yes		emotrepr ⇒ UCL_Depressive	0.23679	0.05323	0.13246	0.3411	0.36756	4.4485	< .001
yes		emotrepr ⇒ UCL_Palliative	0.12366	0.06308	3.55e-5	0.2473	0.18667	1.9605	0.050
yes		emotrepr ⇒ UCL_Avoidant	0.09393	0.06959	-0.04247	0.2303	0.12508	1.3498	0.177
yes		emotrepr ⇒ AvoidanceBehavior	1.09829	0.33311	0.44541	1.7512	0.23613	3.2971	< .001
yes	Direct	timeline ⇒ IBSQoL	0.52622	0.24731	0.04151	1.0109	0.11398	2.1278	0.033
yes		timecycl ⇒ IBSQoL	-0.10261	0.27025	-0.63230	0.4271	-0.01649	-0.3797	0.704
yes		conseque ⇒ IBSQoL	0.56256	0.24290	0.08649	1.0386	0.14609	2.3160	0.021
yes		perscon ⇒ IBSQoL	-0.04453	0.25973	-0.55360	0.4645	-0.00839	-0.1715	0.864
yes		treatcon ⇒ IBSQoL	0.01469	0.27845	-0.53106	0.5604	0.00252	0.0528	0.958
yes		illcoher ⇒ IBSQoL	-0.14958	0.24200	-0.62389	0.3247	-0.03179	-0.6181	0.537
yes		emotrepr ⇒ IBSQoL	1.02596	0.22623	0.58257	1.4694	0.26347	4.5351	< .001
yes	Total	timeline ⇒ IBSQoL	1.03203	0.28439	0.47464	1.5894	0.22333	3.6289	< .001
yes		timecycl ⇒ IBSQoL	-0.13689	0.31992	-0.76391	0.4901	-0.02197	-0.4279	0.669
yes		conseque ⇒ IBSQoL	1.43715	0.26156	0.92450	1.9498	0.37288	5.4945	< .001
yes		perscon ⇒ IBSQoL	-0.12967	0.30871	-0.73473	0.4754	-0.02441	-0.4201	0.674
yes		treatcon ⇒ IBSQoL	-0.23766	0.32594	-0.87650	0.4012	-0.04076	-0.7292	0.466
yes		illcoher ⇒ IBSQoL	-0.19567	0.28580	-0.75582	0.3645	-0.04154	-0.6846	0.494
yes		emotrepr ⇒ IBSQoL	1.67697	0.24949	1.18797	2.1660	0.43026	6.7215	< .001

Note. Confidence intervals computed with method: Standard (Delta method)

Note. Betas are completely standardized effect sizes

[5]

GLM Mediation Analysis

Models Info

Mediators Models		
	m1	UCL_Depressive ~ treatment_seeking + timeline + conseque + emotrepr + timeline:treatment_seeking + conseque:treatment_seeking + emotrepr:treatment_seeking
	m2	AvoidanceBehavior ~ treatment_seeking + timeline + conseque + emotrepr + timeline:treatment_seeking + conseque:treatment_seeking + emotrepr:treatment_seeking
Full Model		
	m3	IBSQoL ~ UCL_Depressive + AvoidanceBehavior + treatment_seeking + timeline + conseque + emotrepr + UCL_Depressive:treatment_seeking + AvoidanceBehavior:treatment_seeking + timeline:treatment_seeking + conseque:treatment_seeking + emotrepr:treatment_seeking
Indirect Effects		
	IE 1	timeline ⇒ UCL_Depressive ⇒ IBSQoL
	IE 2	timeline ⇒ AvoidanceBehavior ⇒ IBSQoL
	IE 3	conseque ⇒ UCL_Depressive ⇒ IBSQoL
	IE 4	conseque ⇒ AvoidanceBehavior ⇒ IBSQoL
	IE 5	emotrepr ⇒ UCL_Depressive ⇒ IBSQoL
	IE 6	emotrepr ⇒ AvoidanceBehavior ⇒ IBSQoL

[3]

Path Model

Model Diagram

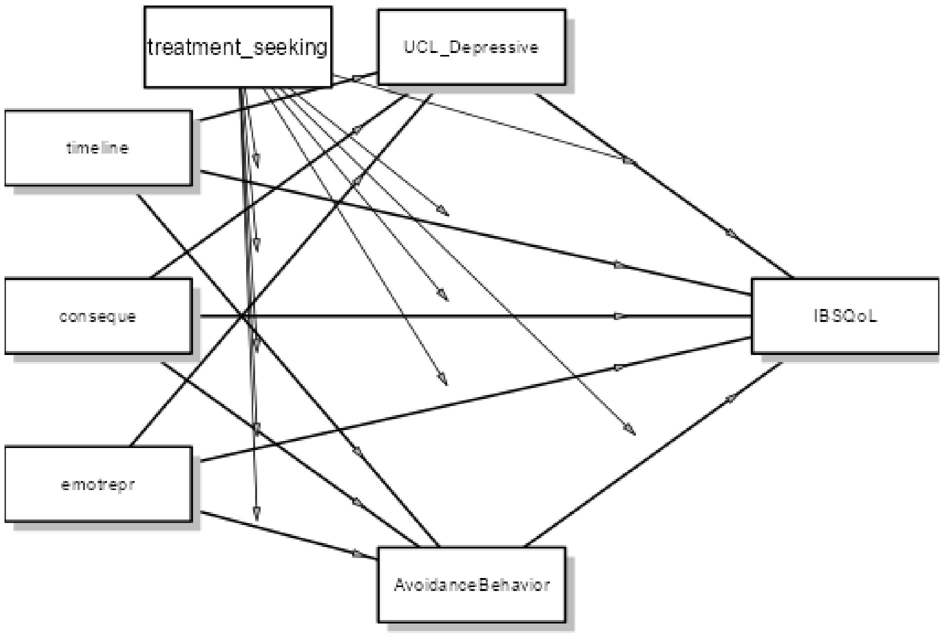

[4]

Model diagram notes
Moderators main effects are not shown
Covariances among IV are estimated but not shown

Mediation

Moderation effects (interactions)

Moderator	Interaction	Estimate	SE	Lower	Upper	β	z	p
	timeline:treatment_seeking1 ⇒ UCL_Depressive	0.1981	0.1074	-0.0124	0.409	0.12498	1.844	0.065
	conseque:treatment_seeking1 ⇒ UCL_Depressive	-0.0465	0.1270	-0.2954	0.202	-0.03529	-0.366	0.714
	emotrepr:treatment_seeking1 ⇒ UCL_Depressive	0.0855	0.1280	-0.1655	0.336	0.06538	0.667	0.505
	timeline:treatment_seeking1 ⇒ AvoidanceBehavior	0.8563	0.6626	-0.4423	2.155	0.07484	1.292	0.196
	conseque:treatment_seeking1 ⇒ AvoidanceBehavior	-0.1447	0.7831	-1.6796	1.390	-0.01521	-0.185	0.853
	emotrepr:treatment_seeking1 ⇒ AvoidanceBehavior	1.0618	0.7897	-0.4861	2.610	0.11254	1.345	0.179
	UCL_Depressive:treatment_seeking1 ⇒ IBSQoL	0.2052	0.5262	-0.8262	1.237	0.05557	0.390	0.697
	AvoidanceBehavior:treatment_seeking1 ⇒ IBSQoL	0.0831	0.0853	-0.0841	0.250	0.07581	0.974	0.330
	timeline:treatment_seeking1 ⇒ IBSQoL	-0.0879	0.4377	-0.9458	0.770	-0.00926	-0.201	0.841
	conseque:treatment_seeking1 ⇒ IBSQoL	0.7503	0.5353	-0.2988	1.799	0.09507	1.402	0.161
	emotrepr:treatment_seeking1 ⇒ IBSQoL	-0.3594	0.5173	-1.3734	0.655	-0.04591	-0.695	0.487

Conditional Mediation

Moderator levels					95% C.I. (a)				
treatment_seeking	Type	Effect	Estimate	SE	Lower	Upper	β	z	p
Average	Indirect	timeline ⇒ UCL_Depressive ⇒ IBSQoL	-0.06466	0.0498	-0.16222	0.03289	-0.01407	-1.2991	0.194
Average		timeline ⇒ AvoidanceBehavior ⇒ IBSQoL	0.35210	0.1173	0.12216	0.58204	0.07661	3.0013	0.003
Average		conseque ⇒ UCL_Depressive ⇒ IBSQoL	0.13658	0.0684	0.00246	0.27071	0.03601	1.9959	0.046
Average		conseque ⇒ AvoidanceBehavior ⇒ IBSQoL	0.71355	0.1579	0.40401	1.02309	0.18811	4.5182	< .001
Average		emotrepr ⇒ UCL_Depressive ⇒ IBSQoL	0.18102	0.0780	0.02822	0.33383	0.04711	2.3219	0.020
Average		emotrepr ⇒ AvoidanceBehavior ⇒ IBSQoL	0.17937	0.1308	-0.07696	0.43570	0.04668	1.3715	0.170
Average	Component	timeline ⇒ UCL_Depressive	-0.07618	0.0537	-0.18146	0.02910	-0.09926	-1.4183	0.156
Average		UCL_Depressive ⇒ IBSQoL	0.84878	0.2621	0.33502	1.36254	0.14173	3.2381	0.001
Average		timeline ⇒ AvoidanceBehavior	1.08053	0.3313	0.43120	1.72986	0.19506	3.2615	0.001
Average		AvoidanceBehavior ⇒ IBSQoL	0.32586	0.0425	0.24257	0.40916	0.39274	7.6675	< .001
Average		conseque ⇒ UCL_Depressive	0.16092	0.0635	0.03649	0.28535	0.25405	2.5347	0.011
Average		conseque ⇒ AvoidanceBehavior	2.18973	0.3916	1.42227	2.95719	0.47897	5.5922	< .001
Average		emotrepr ⇒ UCL_Depressive	0.21327	0.0640	0.08779	0.33876	0.33238	3.3313	< .001
Average		emotrepr ⇒ AvoidanceBehavior	0.55044	0.3949	-0.22350	1.32438	0.11885	1.3940	0.163
Average	Direct	timeline ⇒ IBSQoL	0.57066	0.2183	0.14283	0.99848	0.12416	2.6143	0.009
Average		conseque ⇒ IBSQoL	0.20789	0.2688	-0.31890	0.73468	0.05480	0.7735	0.439
Average		emotrepr ⇒ IBSQoL	1.25782	0.2590	0.75022	1.76541	0.32733	4.8568	< .001
Average	Total	timeline ⇒ IBSQoL	0.88606	0.2484	0.39924	1.37289	0.19067	3.5673	< .001
Average		conseque ⇒ IBSQoL	1.05263	0.2936	0.47724	1.62802	0.27445	3.5856	< .001
Average		emotrepr ⇒ IBSQoL	1.64466	0.2961	1.06441	2.22491	0.42331	5.5553	< .001
no	Indirect	timeline ⇒ UCL_Depressive ⇒ IBSQoL	-0.13077	0.0823	-0.29199	0.03045	-0.02872	-1.5898	0.112
no		timeline ⇒ AvoidanceBehavior ⇒ IBSQoL	0.18547	0.1627	-0.13346	0.50439	0.04074	1.1398	0.254
no		conseque ⇒ UCL_Depressive ⇒ IBSQoL	0.13743	0.0979	-0.05450	0.32935	0.03657	1.4034	0.160
no		conseque ⇒ AvoidanceBehavior ⇒ IBSQoL	0.64310	0.2221	0.20782	1.07838	0.17115	2.8957	0.004
no		emotrepr ⇒ UCL_Depressive ⇒ IBSQoL	0.12726	0.0980	-0.06474	0.31926	0.03343	1.2991	0.194

Note. Confidence intervals computed with method: Standard (Delta method)

Note. Betas are completely standardized effect sizes

[5]

Conditional Mediation

Moderator levels					95% C.I. (a)				
treatment_seeking	Type	Effect	Estimate	SE	Lower	Upper	β	z	p
no		emotrepr ⇒ AvoidanceBehavior ⇒ IBSQoL	0.00555	0.2048	-0.39592	0.40702	0.00146	0.0271	0.978
no	Component	timeline ⇒ UCL_Depressive	-0.17525	0.0914	-0.35447	0.00397	-0.22834	-1.9165	0.055
no		UCL_Depressive ⇒ IBSQoL	0.74619	0.2621	0.23243	1.25995	0.12578	2.8467	0.004
no		timeline ⇒ AvoidanceBehavior	0.65236	0.5640	-0.45304	1.75777	0.11777	1.1567	0.247
no		AvoidanceBehavior ⇒ IBSQoL	0.28430	0.0425	0.20100	0.36760	0.34589	6.6895	< .001
no		conseque ⇒ UCL_Depressive	0.18417	0.1142	-0.03960	0.40794	0.29076	1.6131	0.107
no		conseque ⇒ AvoidanceBehavior	2.26206	0.7042	0.88187	3.64225	0.49479	3.2123	0.001
no		emotrepr ⇒ UCL_Depressive	0.17055	0.1168	-0.05841	0.39950	0.26579	1.4600	0.144
no		emotrepr ⇒ AvoidanceBehavior	0.01953	0.7205	-1.39261	1.43167	0.00422	0.0271	0.978
no	Direct	timeline ⇒ IBSQoL	0.61462	0.3619	-0.09476	1.32401	0.13499	1.6982	0.089
no		conseque ⇒ IBSQoL	-0.16728	0.4597	-1.06823	0.73368	-0.04452	-0.3639	0.716
no		emotrepr ⇒ IBSQoL	1.43751	0.4594	0.53703	2.33800	0.37765	3.1288	0.002
no	Total	timeline ⇒ IBSQoL	0.66932	0.4228	-0.15944	1.49808	0.14403	1.5829	0.113
no		conseque ⇒ IBSQoL	0.61325	0.5280	-0.42152	1.64803	0.15989	1.1616	0.245
no		emotrepr ⇒ IBSQoL	1.57032	0.5402	0.51159	2.62905	0.40417	2.9070	0.004
yes	Indirect	timeline ⇒ UCL_Depressive ⇒ IBSQoL	0.02177	0.0540	-0.08402	0.12757	0.00468	0.4034	0.687
yes		timeline ⇒ AvoidanceBehavior ⇒ IBSQoL	0.55434	0.1430	0.27413	0.83454	0.11913	3.8774	< .001
yes		conseque ⇒ UCL_Depressive ⇒ IBSQoL	0.13097	0.0640	0.00553	0.25640	0.03410	2.0464	0.041
yes		conseque ⇒ AvoidanceBehavior ⇒ IBSQoL	0.77799	0.1548	0.47467	1.08130	0.20258	5.0272	< .001
yes		emotrepr ⇒ UCL_Depressive ⇒ IBSQoL	0.24356	0.0836	0.07967	0.40744	0.06261	2.9128	0.004
yes		emotrepr ⇒ AvoidanceBehavior ⇒ IBSQoL	0.39732	0.1274	0.14761	0.64703	0.10213	3.1185	0.002
yes	Component	timeline ⇒ UCL_Depressive	0.02289	0.0564	-0.08763	0.13340	0.02982	0.4059	0.685
yes		UCL_Depressive ⇒ IBSQoL	0.95138	0.2621	0.43762	1.46514	0.15692	3.6295	< .001
yes		timeline ⇒ AvoidanceBehavior	1.50870	0.3478	0.82709	2.19032	0.27236	4.3382	< .001
yes		AvoidanceBehavior ⇒ IBSQoL	0.36743	0.0425	0.28413	0.45072	0.43741	8.6455	< .001
yes		conseque ⇒ UCL_Depressive	0.13766	0.0556	0.02877	0.24655	0.21734	2.4779	0.013
yes		conseque ⇒ AvoidanceBehavior	2.11739	0.3427	1.44578	2.78900	0.46314	6.1792	< .001

Note. Confidence intervals computed with method: Standard (Delta method)

Note. Betas are completely standardized effect sizes

Conditional Mediation

Moderator levels					95% C.I. (a)				
**treatment_seeking**	**Type**	**Effect**	**Estimate**	**SE**	**Lower**	**Upper**	**β**	**z**	**p**
yes		emotrepr ⇒ UCL_Depressive	0.25600	0.0524	0.15323	0.35877	0.39898	4.8823	< .001
yes		emotrepr ⇒ AvoidanceBehavior	1.08135	0.3234	0.44749	1.71522	0.23349	3.3436	< .001
yes	Direct	timeline ⇒ IBSQoL	0.52669	0.2299	0.07607	0.97732	0.11319	2.2908	0.022
yes		conseque ⇒ IBSQoL	0.58305	0.2381	0.11638	1.04973	0.15182	2.4487	0.014
yes		emotrepr ⇒ IBSQoL	1.07813	0.2208	0.64541	1.51084	0.27713	4.8833	< .001
yes	Total	timeline ⇒ IBSQoL	1.10280	0.2607	0.59177	1.61383	0.23731	4.2296	< .001
yes		conseque ⇒ IBSQoL	1.49201	0.2569	0.98848	1.99553	0.38901	5.8076	< .001
yes		emotrepr ⇒ IBSQoL	1.71900	0.2425	1.24377	2.19423	0.44244	7.0896	< .001

Note. Confidence intervals computed with method: Standard (Delta method)

Note. Betas are completely standardized effect sizes

[5]

References

[1] The jamovi project (2021). *jamovi*. (Version 2.2) [Computer Software]. Retrieved from https://www.jamovi.org.

[2] R Core Team (2021). *R: A Language and environment for statistical computing*. (Version 4.0) [Computer software]. Retrieved from https://cran.r-project.org. (R packages retrieved from MRAN snapshot 2021-04-01).

[3] Gallucci, M. (2020). *jAMM: jamovi Advanced Mediation Models*. [jamovi module]. Retrieved from https://jamovi-amm.github.io/.

[4] Soetaert, K. (2019). *diagram: Functions for Visualising Simple Graphs (Networks), Plotting Flow Diagrams*. [R package]. Retrieved from https://cran.r-project.org/package=diagram.

[5] Rosseel, Y. (2019). lavaan: An R Package for Structural Equation Modeling. *Journal of Statistical Software, 48(2), 1-36. link*.

Supplement II: Analysis output for moderated mediation model, including all variables and output of simplified moderated mediation model withROME III filter

Results

GLM Mediation Analysis

Mediators Models		
	m1	UCL_Depressive ~ treatment_seeking + timeline + timecycl + conseque + perscon + treatcon + illcoher + emotrepr + timeline:treatment_seeking + timecycl:treatment_seeking + conseque:treatment_seeking + perscon:treatment_seeking + treatcon:treatment_seeking + illcoher:treatment_seeking + emotrepr:treatment_seeking
	m2	UCL_Palliative ~ treatment_seeking + timeline + timecycl + conseque + perscon + treatcon + illcoher + emotrepr + timeline:treatment_seeking + timecycl:treatment_seeking + conseque:treatment_seeking + perscon:treatment_seeking + treatcon:treatment_seeking + illcoher:treatment_seeking + emotrepr:treatment_seeking
	m3	UCL_Avoidant ~ treatment_seeking + timeline + timecycl + conseque + perscon + treatcon + illcoher + emotrepr + timeline:treatment_seeking + timecycl:treatment_seeking + conseque:treatment_seeking + perscon:treatment_seeking + treatcon:treatment_seeking + illcoher:treatment_seeking + emotrepr:treatment_seeking
	m4	AvoidanceBehavior ~ treatment_seeking + timeline + timecycl + conseque + perscon + treatcon + illcoher + emotrepr + timeline:treatment_seeking + timecycl:treatment_seeking + conseque:treatment_seeking + perscon:treatment_seeking + treatcon:treatment_seeking + illcoher:treatment_seeking + emotrepr:treatment_seeking
Full Model		
	m5	IBSQoL ~ UCL_Depressive + UCL_Palliative + UCL_Avoidant + AvoidanceBehavior + treatment_seeking + timeline + timecycl + conseque + perscon + treatcon + illcoher + emotrepr + timeline:treatment_seeking + timecycl:treatment_seeking + conseque:treatment_seeking + perscon:treatment_seeking + treatcon:treatment_seeking + illcoher:treatment_seeking + emotrepr:treatment_seeking + treatment_seeking:UCL_Depressive + treatment_seeking:UCL_Palliative + treatment_seeking:UCL_Avoidant + treatment_seeking:AvoidanceBehavior
Indirect Effects		
	IE 1	timeline ⇒ UCL_Depressive ⇒ IBSQoL
	IE 2	timeline ⇒ UCL_Palliative ⇒ IBSQoL
	IE 3	timeline ⇒ UCL_Avoidant ⇒ IBSQoL
	IE 4	timeline ⇒ AvoidanceBehavior ⇒ IBSQoL
	IE 5	timecycl ⇒ UCL_Depressive ⇒ IBSQoL
	IE 6	timecycl ⇒ UCL_Palliative ⇒ IBSQoL
	IE 7	timecycl ⇒ UCL_Avoidant ⇒ IBSQoL
	IE 8	timecycl ⇒ AvoidanceBehavior ⇒ IBSQoL
	IE 9	conseque ⇒ UCL_Depressive ⇒ IBSQoL
	IE 10	conseque ⇒ UCL_Palliative ⇒ IBSQoL
	IE 11	conseque ⇒ UCL_Avoidant ⇒ IBSQoL
	IE 12	conseque ⇒ AvoidanceBehavior ⇒ IBSQoL
	IE 13	perscon ⇒ UCL_Depressive ⇒ IBSQoL
	IE 14	perscon ⇒ UCL_Palliative ⇒ IBSQoL
	IE 15	perscon ⇒ UCL_Avoidant ⇒ IBSQoL
	IE 16	perscon ⇒ AvoidanceBehavior ⇒ IBSQoL
	IE 17	treatcon ⇒ UCL_Depressive ⇒ IBSQoL
	IE 18	treatcon ⇒ UCL_Palliative ⇒ IBSQoL

IE 19	treatcon ⇒ UCL_Avoidant ⇒ IBSQoL
IE 20	treatcon ⇒ AvoidanceBehavior ⇒ IBSQoL
IE 21	illcoher ⇒ UCL_Depressive ⇒ IBSQoL
IE 22	illcoher ⇒ UCL_Palliative ⇒ IBSQoL
IE 23	illcoher ⇒ UCL_Avoidant ⇒ IBSQoL
IE 24	illcoher ⇒ AvoidanceBehavior ⇒ IBSQoL
IE 25	emotrepr ⇒ UCL_Depressive ⇒ IBSQoL
IE 26	emotrepr ⇒ UCL_Palliative ⇒ IBSQoL
IE 27	emotrepr ⇒ UCL_Avoidant ⇒ IBSQoL
IE 28	emotrepr ⇒ AvoidanceBehavior ⇒ IBSQoL

[3]

Path Model

Model Diagram

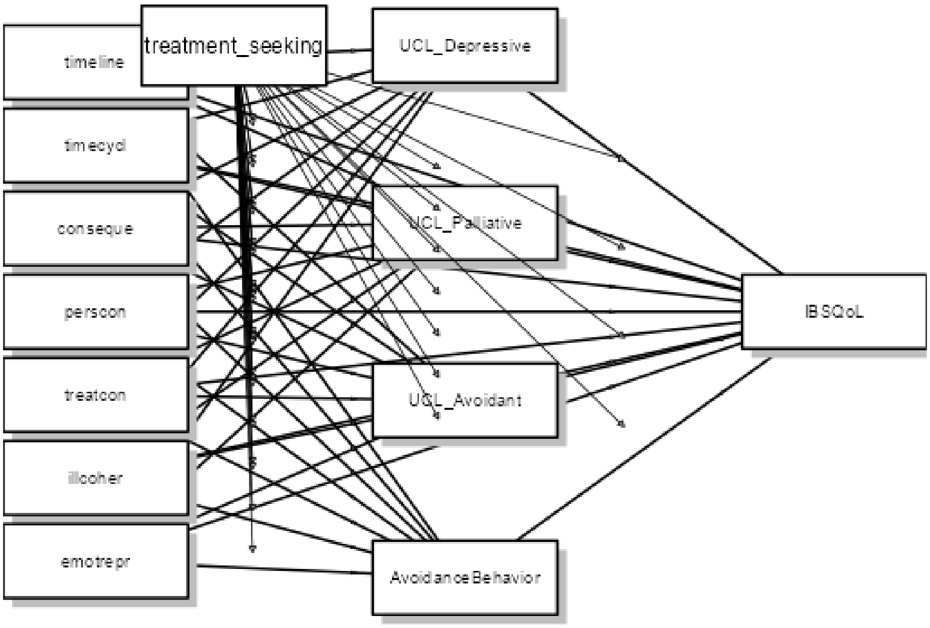

[4]

Model diagram notes
Moderators main effects are not shown
Covariances among IV are estimated but not shown

Mediation

Moderation effects (interactions)

Moderator	Interaction	Estimate	SE	Lower	Upper	β	z	p
	timeline:treatment_seeking1 ⇒ UCL_Depressive	0.19446	0.170	-0.1395	0.5285	0.11778	1.1411	0.254
	timecycl:treatment_seeking1 ⇒ UCL_Depressive	-0.17258	0.224	-0.6107	0.2656	-0.08001	-0.7720	0.440
	conseque:treatment_seeking1 ⇒ UCL_Depressive	-0.29292	0.209	-0.7018	0.1159	-0.20936	-1.4042	0.160
	perscon:treatment_seeking1 ⇒ UCL_Depressive	-0.00747	0.155	-0.3109	0.2960	-0.00399	-0.0482	0.962
	treatcon:treatment_seeking1 ⇒ UCL_Depressive	-0.31986	0.228	-0.7661	0.1264	-0.16126	-1.4050	0.160
	illcoher:treatment_seeking1 ⇒ UCL_Depressive	-0.16684	0.157	-0.4745	0.1408	-0.10430	-1.0629	0.288
	emotrepr:treatment_seeking1 ⇒ UCL_Depressive	0.44455	0.227	5.27e-4	0.8886	0.28856	1.9623	0.050
	timeline:treatment_seeking1 ⇒ UCL_Palliative	-0.41867	0.191	-0.7927	-0.0446	-0.26205	-2.1936	0.028
	timecycl:treatment_seeking1 ⇒ UCL_Palliative	-0.21438	0.250	-0.7051	0.2764	-0.10271	-0.8562	0.392
	conseque:treatment_seeking1 ⇒ UCL_Palliative	0.18877	0.234	-0.2691	0.6467	0.13942	0.8080	0.419
	perscon:treatment_seeking1 ⇒ UCL_Palliative	0.00205	0.173	-0.3378	0.3419	0.00113	0.0118	0.991
	treatcon:treatment_seeking1 ⇒ UCL_Palliative	-0.16512	0.255	-0.6649	0.3346	-0.08603	-0.6476	0.517
	illcoher:treatment_seeking1 ⇒ UCL_Palliative	-0.06768	0.176	-0.4122	0.2769	-0.04372	-0.3849	0.700
	emotrepr:treatment_seeking1 ⇒ UCL_Palliative	-0.14026	0.254	-0.6376	0.3570	-0.09408	-0.5528	0.580
	timeline:treatment_seeking1 ⇒ UCL_Avoidant	-0.39969	0.221	-0.8319	0.0325	-0.20901	-1.8124	0.070
	timecycl:treatment_seeking1 ⇒ UCL_Avoidant	0.35299	0.289	-0.2140	0.9200	0.14129	1.2201	0.222
	conseque:treatment_seeking1 ⇒ UCL_Avoidant	0.15559	0.270	-0.3735	0.6847	0.09601	0.5764	0.564
	perscon:treatment_seeking1 ⇒ UCL_Avoidant	0.16836	0.200	-0.2243	0.5610	0.07765	0.8404	0.401
	treatcon:treatment_seeking1 ⇒ UCL_Avoidant	-0.52598	0.295	-1.1034	0.0515	-0.22894	-1.7852	0.074
	illcoher:treatment_seeking1 ⇒ UCL_Avoidant	-0.03682	0.203	-0.4350	0.3613	-0.01987	-0.1813	0.856
	emotrepr:treatment_seeking1 ⇒ UCL_Avoidant	-0.06754	0.293	-0.6422	0.5071	-0.03785	-0.2304	0.818
	timeline:treatment_seeking1 ⇒ AvoidanceBehavior	0.63478	1.033	-1.3907	2.6602	0.05685	0.6143	0.539
	timecycl:treatment_seeking1 ⇒ AvoidanceBehavior	-1.73091	1.356	-4.3880	0.9262	-0.11866	-1.2768	0.202
	conseque:treatment_seeking1 ⇒ AvoidanceBehavior	-0.41415	1.265	-2.8935	2.0652	-0.04377	-0.3274	0.743
	perscon:treatment_seeking1 ⇒ AvoidanceBehavior	-0.67168	0.939	-2.5117	1.1684	-0.05306	-0.7154	0.474
	treatcon:treatment_seeking1 ⇒ AvoidanceBehavior	0.82368	1.381	-1.8823	3.5297	0.06140	0.5966	0.551
	illcoher:treatment_seeking1 ⇒ AvoidanceBehavior	-1.95104	0.952	-3.8167	-0.0854	-0.18035	-2.0496	0.040
	emotrepr:treatment_seeking1 ⇒ AvoidanceBehavior	0.76177	1.374	-1.9309	3.4545	0.07312	0.5545	0.579
	timeline:treatment_seeking1 ⇒ IBSQoL	-0.09296	0.659	-1.3846	1.1987	-0.01092	-0.1411	0.888
	timecycl:treatment_seeking1 ⇒ IBSQoL	1.39932	0.824	-0.2164	3.0150	0.12584	1.6975	0.090
	conseque:treatment_seeking1 ⇒ IBSQoL	1.21556	0.811	-0.3740	2.8051	0.16852	1.4989	0.134
	perscon:treatment_seeking1 ⇒ IBSQoL	-0.10107	0.565	-1.2082	1.0061	-0.01047	-0.1789	0.858
	treatcon:treatment_seeking1 ⇒ IBSQoL	-0.09248	0.858	-1.7745	1.5895	-0.00904	-0.1078	0.914
	illcoher:treatment_seeking1 ⇒ IBSQoL	0.44282	0.588	-0.7088	1.5945	0.05370	0.7536	0.451
	emotrepr:treatment_seeking1 ⇒ IBSQoL	-0.40492	0.836	-2.0443	1.2345	-0.05098	-0.4841	0.628
	treatment_seeking1:UCL_Depressive ⇒ IBSQoL	1.25849	0.634	0.0162	2.5008	0.38136	1.9856	0.047
	treatment_seeking1:UCL_Palliative ⇒ IBSQoL	0.48038	0.541	-0.5807	1.5415	0.17348	0.8873	0.375
	treatment_seeking1:UCL_Avoidant ⇒ IBSQoL	-0.09224	0.492	-1.0566	0.8721	-0.04145	-0.1875	0.851
	treatment_seeking1:AvoidanceBehavior ⇒ IBSQoL	0.01738	0.101	-0.1808	0.2155	0.01842	0.1719	0.864

Conditional Mediation

Moderator levels					95% C.I. (a)				
treatment_seeking	Type	Effect	Estimate	SE	Lower	Upper	β	z	p
Average	Indirect	timeline ⇒ UCL_Depressive ⇒ IBSQoL	-0.04864	0.05134	-0.14925	0.05198	-0.01154	-0.94744	0.343
Average		timeline ⇒ UCL_Palliative ⇒ IBSQoL	-0.00746	0.06675	-0.13829	0.12338	-0.00177	-0.11170	0.911
Average		timeline ⇒ UCL_Avoidant ⇒ IBSQoL	-0.00851	0.03015	-0.06760	0.05058	-0.00202	-0.28233	0.778
Average		timeline ⇒ AvoidanceBehavior ⇒ IBSQoL	0.20399	0.14541	-0.08100	0.48898	0.04840	1.40291	0.161
Average		timecycl ⇒ UCL_Depressive ⇒ IBSQoL	0.01165	0.05538	-0.09690	0.12020	0.00210	0.21034	0.833
Average		timecycl ⇒ UCL_Palliative ⇒ IBSQoL	2.28e-4	0.00427	-0.00814	0.00860	4.11e-5	0.05350	0.957
Average		timecycl ⇒ UCL_Avoidant ⇒ IBSQoL	-4.64e−5	0.00978	-0.01921	0.01911	-8.35e−6	-0.00475	0.996
Average		timecycl ⇒ AvoidanceBehavior ⇒ IBSQoL	0.22314	0.18885	-0.14700	0.59328	0.04015	1.18157	0.237
Average		conseque ⇒ UCL_Depressive ⇒ IBSQoL	0.13575	0.09747	-0.05530	0.32680	0.03810	1.39268	0.164
Average		conseque ⇒ UCL_Palliative ⇒ IBSQoL	0.00436	0.03915	-0.07237	0.08109	0.00122	0.11135	0.911
Average		conseque ⇒ UCL_Avoidant ⇒ IBSQoL	-0.01015	0.03599	-0.08069	0.06040	-0.00285	-0.28186	0.778
Average		conseque ⇒ AvoidanceBehavior ⇒ IBSQoL	0.64350	0.20813	0.23558	1.05143	0.18061	3.09184	0.002
Average		perscon ⇒ UCL_Depressive ⇒ IBSQoL	4.45e-4	0.03804	-0.07410	0.07499	9.24e-5	0.01171	0.991
Average		perscon ⇒ UCL_Palliative ⇒ IBSQoL	-0.00191	0.01728	-0.03579	0.03197	-3.96e−4	-0.11053	0.912
Average		perscon ⇒ UCL_Avoidant ⇒ IBSQoL	0.00523	0.01919	-0.03238	0.04285	0.00109	0.27264	0.785
Average		perscon ⇒ AvoidanceBehavior ⇒ IBSQoL	-0.07418	0.12844	-0.32591	0.17755	-0.01539	-0.57760	0.564
Average		treatcon ⇒ UCL_Depressive ⇒ IBSQoL	0.02776	0.05845	-0.08679	0.14231	0.00546	0.47494	0.635
Average		treatcon ⇒ UCL_Palliative ⇒ IBSQoL	-0.00714	0.06397	-0.13252	0.11824	-0.00140	-0.11160	0.911
Average		treatcon ⇒ UCL_Avoidant ⇒ IBSQoL	-0.00869	0.03143	-0.07029	0.05292	-0.00171	-0.27637	0.782

Note. Confidence intervals computed with method: Standard (Delta method)

Note. Betas are completely standardized effect sizes

[5]

Conditional Mediation

Moderator levels					95% C.I. (a)				
treatment_seeking	Type	Effect	Estimate	SE	Lower	Upper	β	z	p
Average		treatcon ⇒ AvoidanceBehavior ⇒ IBSQoL	-0.25854	0.19363	-0.63804	0.12097	-0.05085	-1.33523	0.182
Average		illcoher ⇒ UCL_Depressive ⇒ IBSQoL	-0.00613	0.03875	-0.08207	0.06981	-0.00149	-0.15822	0.874
Average		illcoher ⇒ UCL_Palliative ⇒ IBSQoL	-0.00207	0.01871	-0.03873	0.03459	-5.02e−4	-0.11069	0.912
Average		illcoher ⇒ UCL_Avoidant ⇒ IBSQoL	-0.00255	0.01112	-0.02434	0.01924	-6.18e−4	-0.22923	0.819
Average		illcoher ⇒ AvoidanceBehavior ⇒ IBSQoL	0.26960	0.13847	-0.00181	0.54100	0.06538	1.94690	0.052
Average		emotrepr ⇒ UCL_Depressive ⇒ IBSQoL	0.02402	0.05756	-0.08879	0.13684	0.00607	0.41738	0.676
Average		emotrepr ⇒ UCL_Palliative ⇒ IBSQoL	-0.00389	0.03498	-0.07245	0.06468	-9.82e−4	-0.11114	0.912
Average		emotrepr ⇒ UCL_Avoidant ⇒ IBSQoL	-0.00574	0.02204	-0.04893	0.03745	-0.00145	-0.26027	0.795
Average		emotrepr ⇒ AvoidanceBehavior ⇒ IBSQoL	0.14110	0.18866	-0.22867	0.51087	0.03562	0.74790	0.455
Average	Component	timeline ⇒ UCL_Depressive	-0.09898	0.08521	-0.26598	0.06802	-0.12109	-1.16171	0.245
Average		UCL_Depressive ⇒ IBSQoL	0.49136	0.30012	-0.09685	1.07958	0.09531	1.63725	0.102
Average		timeline ⇒ UCL_Palliative	0.24889	0.09543	0.06185	0.43592	0.31463	2.60806	0.009
Average		UCL_Palliative ⇒ IBSQoL	-0.02996	0.26796	-0.55515	0.49524	-0.00562	-0.11180	0.911
Average		timeline ⇒ UCL_Avoidant	0.12597	0.11026	-0.09015	0.34208	0.13304	1.14242	0.253
Average		UCL_Avoidant ⇒ IBSQoL	-0.06757	0.23191	-0.52210	0.38696	-0.01518	-0.29137	0.771
Average		timeline ⇒ AvoidanceBehavior	0.74971	0.51671	-0.26301	1.76243	0.13562	1.45094	0.147
Average		AvoidanceBehavior ⇒ IBSQoL	0.27210	0.04949	0.17510	0.36909	0.35692	5.49808	< .001
Average		timecycl ⇒ UCL_Depressive	0.02371	0.11178	-0.19537	0.24279	0.02199	0.21210	0.832
Average		timecycl ⇒ UCL_Palliative	-0.00763	0.12519	-0.25299	0.23774	-0.00731	-0.06092	0.951
Average		timecycl ⇒ UCL_Avoidant	6.86e-4	0.14465	-0.28283	0.28420	5.50e-4	0.00475	0.996
Average		timecycl ⇒ AvoidanceBehavior	0.82008	0.67784	-0.50847	2.14863	0.11248	1.20984	0.226
Average		conseque ⇒ UCL_Depressive	0.27627	0.10430	0.07185	0.48070	0.39975	2.64881	0.008
Average		conseque ⇒ UCL_Palliative	-0.14552	0.11682	-0.37448	0.08343	-0.21759	-1.24574	0.213
Average		conseque ⇒ UCL_Avoidant	0.15014	0.13498	-0.11441	0.41469	0.18756	1.11235	0.266

Note. Confidence intervals computed with method: Standard (Delta method)

Note. Betas are completely standardized effect sizes

[5]

Conditional Mediation

Moderator levels					95% C.I. (a)				
treatment_seeking	Type	Effect	Estimate	SE	Lower	Upper	β	z	p
Average		conseque ⇒ AvoidanceBehavior	2.36498	0.63250	1.12529	3.60466	0.50601	3.73907	< .001
Average		perscon ⇒ UCL_Depressive	9.07e-4	0.07741	-0.15081	0.15262	9.69e-4	0.01171	0.991
Average		perscon ⇒ UCL_Palliative	0.06377	0.08669	-0.10615	0.23369	0.07046	0.73560	0.462
Average		perscon ⇒ UCL_Avoidant	-0.07744	0.10017	-0.27377	0.11889	-0.07148	-0.77307	0.439
Average		perscon ⇒ AvoidanceBehavior	-0.27264	0.46941	-1.19267	0.64739	-0.04310	-0.58081	0.561
Average		treatcon ⇒ UCL_Depressive	0.05649	0.11383	-0.16662	0.27960	0.05729	0.49628	0.620
Average		treatcon ⇒ UCL_Palliative	0.23831	0.12749	-0.01157	0.48819	0.24973	1.86923	0.062
Average		treatcon ⇒ UCL_Avoidant	0.12856	0.14731	-0.16017	0.41728	0.11255	0.87269	0.383
Average		treatcon ⇒ AvoidanceBehavior	-0.95017	0.69031	-2.30316	0.40282	-0.14248	-1.37643	0.169
Average		illcoher ⇒ UCL_Depressive	-0.01248	0.07848	-0.16630	0.14135	-0.01560	-0.15896	0.874
Average		illcoher ⇒ UCL_Palliative	0.06911	0.08790	-0.10317	0.24140	0.08930	0.78626	0.432
Average		illcoher ⇒ UCL_Avoidant	0.03772	0.10157	-0.16135	0.23678	0.04072	0.37136	0.710
Average		illcoher ⇒ AvoidanceBehavior	0.99082	0.47594	0.05798	1.92365	0.18319	2.08179	0.037
Average		emotrepr ⇒ UCL_Depressive	0.04889	0.11327	-0.17312	0.27091	0.06364	0.43165	0.666
Average		emotrepr ⇒ UCL_Palliative	0.12978	0.12687	-0.11887	0.37843	0.17457	1.02298	0.306
Average		emotrepr ⇒ UCL_Avoidant	0.08488	0.14659	-0.20243	0.37219	0.09539	0.57905	0.563
Average		emotrepr ⇒ AvoidanceBehavior	0.51856	0.68692	-0.82777	1.86490	0.09981	0.75491	0.450
Average	Direct	timeline ⇒ IBSQoL	0.63567	0.33040	-0.01190	1.28323	0.15083	1.92394	0.054
Average		timecycl ⇒ IBSQoL	-0.36224	0.41427	-1.17419	0.44972	-0.06517	-0.87440	0.382
Average		conseque ⇒ IBSQoL	0.29656	0.41078	-0.50855	1.10167	0.08323	0.72195	0.470
Average		perscon ⇒ IBSQoL	-0.36439	0.28262	-0.91831	0.18953	-0.07557	-1.28934	0.197
Average		treatcon ⇒ IBSQoL	0.04881	0.42791	-0.78987	0.88749	0.00960	0.11408	0.909
Average		illcoher ⇒ IBSQoL	-0.48663	0.29351	-1.06190	0.08863	-0.11802	-1.65799	0.097
Average		emotrepr ⇒ IBSQoL	0.90653	0.41900	0.08530	1.72776	0.22888	2.16355	0.030
Average	Total	timeline ⇒ IBSQoL	0.79786	0.35765	0.09687	1.49885	0.18692	2.23080	0.026
Average		timecycl ⇒ IBSQoL	-0.22300	0.46919	-1.14260	0.69660	-0.03961	-0.47529	0.635
Average		conseque ⇒ IBSQoL	0.99520	0.43781	0.13712	1.85329	0.27578	2.27315	0.023
Average		perscon ⇒ IBSQoL	-0.44368	0.32492	-1.08051	0.19315	-0.09085	-1.36551	0.172
Average		treatcon ⇒ IBSQoL	-0.30259	0.47782	-1.23911	0.63392	-0.05876	-0.63327	0.527
Average		illcoher ⇒ IBSQoL	-0.29602	0.32944	-0.94171	0.34967	-0.07088	-0.89854	0.369
Average		emotrepr ⇒ IBSQoL	1.18985	0.47547	0.25794	2.12176	0.29660	2.50244	0.012
no	Indirect	timeline ⇒ UCL_Depressive ⇒ IBSQoL	0.02691	0.06253	-0.09566	0.14947	0.00633	0.43031	0.667
no		timeline ⇒ UCL_Palliative ⇒ IBSQoL	-0.12386	0.13128	-0.38116	0.13344	-0.02913	-0.94351	0.345

Note. Confidence intervals computed with method: Standard (Delta method)

Note. Betas are completely standardized effect sizes

[5]

Conditional Mediation

Moderator levels					95% C.I. (a)				
treatment_seeking	Type	Effect	Estimate	SE	Lower	Upper	β	z	p
no		timeline ⇒ UCL_Avoidant ⇒ IBSQoL	-0.00716	0.07568	-0.15550	0.14117	-0.00168	-0.09465	0.925
no		timeline ⇒ AvoidanceBehavior ⇒ IBSQoL	0.11434	0.24612	-0.36804	0.59672	0.02689	0.46456	0.642
no		timecycl ⇒ UCL_Depressive ⇒ IBSQoL	-0.01508	0.04347	-0.10029	0.07012	-0.00269	-0.34698	0.729
no		timecycl ⇒ UCL_Palliative ⇒ IBSQoL	-0.02692	0.06791	-0.16002	0.10618	-0.00480	-0.39638	0.692
no		timecycl ⇒ UCL_Avoidant ⇒ IBSQoL	0.00387	0.04120	-0.07688	0.08461	6.89e-4	0.09384	0.925
no		timecycl ⇒ AvoidanceBehavior ⇒ IBSQoL	0.44457	0.34002	-0.22186	1.11100	0.07929	1.30747	0.191
no		conseque ⇒ UCL_Depressive ⇒ IBSQoL	-0.05797	0.12971	-0.31219	0.19625	-0.01613	-0.44696	0.655
no		conseque ⇒ UCL_Palliative ⇒ IBSQoL	0.06485	0.08763	-0.10691	0.23660	0.01804	0.73999	0.459
no		conseque ⇒ UCL_Avoidant ⇒ IBSQoL	-0.00159	0.01768	-0.03625	0.03307	-4.42e−4	-0.08993	0.928
no		conseque ⇒ AvoidanceBehavior ⇒ IBSQoL	0.67757	0.33913	0.01288	1.34225	0.18851	1.99795	0.046
no		perscon ⇒ UCL_Depressive ⇒ IBSQoL	-6.36e−4	0.01763	-0.03519	0.03392	-1.31e−4	-0.03609	0.971
no		perscon ⇒ UCL_Palliative ⇒ IBSQoL	-0.01696	0.04229	-0.09984	0.06592	-0.00349	-0.40107	0.688
no		perscon ⇒ UCL_Avoidant ⇒ IBSQoL	0.00355	0.03766	-0.07026	0.07737	7.31e-4	0.09436	0.925
no		perscon ⇒ AvoidanceBehavior ⇒ IBSQoL	0.01669	0.20482	-0.38475	0.41814	0.00343	0.08150	0.935
no		treatcon ⇒ UCL_Depressive ⇒ IBSQoL	-0.02968	0.07119	-0.16920	0.10985	-0.00579	-0.41692	0.677
no		treatcon ⇒ UCL_Palliative ⇒ IBSQoL	-0.08674	0.10739	-0.29721	0.12373	-0.01691	-0.80774	0.419
no		treatcon ⇒ UCL_Avoidant ⇒ IBSQoL	-0.00861	0.09100	-0.18696	0.16974	-0.00168	-0.09459	0.925
no		treatcon ⇒ AvoidanceBehavior ⇒ IBSQoL	-0.35811	0.34614	-1.03653	0.32031	-0.06983	-1.03459	0.301
no		illcoher ⇒ UCL_Depressive ⇒ IBSQoL	-0.00973	0.02815	-0.06490	0.04545	-0.00234	-0.34560	0.730

Note. Confidence intervals computed with method: Standard (Delta method)

Note. Betas are completely standardized effect sizes

[5]

Moderator levels					95% C.I. (a)				
treatment_seeking	Type	Effect	Estimate	SE	Lower	Upper	β	z	p
no		illcoher ⇒ UCL_Palliative ⇒ IBSQoL	-0.02783	0.04913	-0.12413	0.06847	-0.00669	-0.56640	0.571
no		illcoher ⇒ UCL_Avoidant ⇒ IBSQoL	-0.00123	0.01357	-0.02782	0.02536	-2.97e−4	-0.09097	0.928
no		illcoher ⇒ AvoidanceBehavior ⇒ IBSQoL	0.51798	0.23561	0.05619	0.97978	0.12453	2.19845	0.028
no		emotrepr ⇒ UCL_Depressive ⇒ IBSQoL	0.02378	0.05965	-0.09313	0.14068	0.00595	0.39865	0.690
no		emotrepr ⇒ UCL_Palliative ⇒ IBSQoL	-0.05403	0.08373	-0.21815	0.11008	-0.01352	-0.64530	0.519
no		emotrepr ⇒ UCL_Avoidant ⇒ IBSQoL	-0.00261	0.02818	-0.05784	0.05262	-6.53e−4	-0.09259	0.926
no		emotrepr ⇒ AvoidanceBehavior ⇒ IBSQoL	0.03640	0.33978	-0.62955	0.70236	0.00911	0.10713	0.915
no	Component	timeline ⇒ UCL_Depressive	-0.19622	0.15343	-0.49694	0.10450	-0.24003	-1.27885	0.201
no		UCL_Depressive ⇒ IBSQoL	-0.13714	0.30012	-0.72535	0.45108	-0.02637	-0.45695	0.648
no		timeline ⇒ UCL_Palliative	0.45822	0.17184	0.12141	0.79502	0.57925	2.66648	0.008
no		UCL_Palliative ⇒ IBSQoL	-0.27031	0.26796	-0.79551	0.25488	-0.05030	-1.00877	0.313
no		timeline ⇒ UCL_Avoidant	0.32581	0.19856	-0.06336	0.71497	0.34410	1.64087	0.101
no		UCL_Avoidant ⇒ IBSQoL	-0.02199	0.23191	-0.47652	0.43255	-0.00490	-0.09480	0.924
no		timeline ⇒ AvoidanceBehavior	0.43391	0.93045	-1.38974	2.25756	0.07849	0.46634	0.641
no		AvoidanceBehavior ⇒ IBSQoL	0.26350	0.04949	0.16651	0.36050	0.34264	5.32448	< .001
no		timecycl ⇒ UCL_Depressive	0.10999	0.20627	-0.29429	0.51427	0.10201	0.53323	0.594
no		timecycl ⇒ UCL_Palliative	0.09958	0.23102	-0.35321	0.55237	0.09545	0.43105	0.666
no		timecycl ⇒ UCL_Avoidant	-0.17583	0.26694	-0.69902	0.34735	-0.14080	-0.65872	0.510
no		timecycl ⇒ AvoidanceBehavior	1.68713	1.25087	-0.76452	4.13879	0.23139	1.34877	0.177
no		conseque ⇒ UCL_Depressive	0.42274	0.19672	0.03717	0.80830	0.61168	2.14893	0.032
no		conseque ⇒ UCL_Palliative	-0.23990	0.22033	-0.67173	0.19193	-0.35870	-1.08883	0.276
no		conseque ⇒ UCL_Avoidant	0.07233	0.25458	-0.42664	0.57129	0.09035	0.28411	0.776
no		conseque ⇒ AvoidanceBehavior	2.57137	1.19296	0.23322	4.90953	0.55017	2.15546	0.031
no		perscon ⇒ UCL_Depressive	0.00464	0.12816	-0.24655	0.25584	0.00496	0.03621	0.971
no		perscon ⇒ UCL_Palliative	0.06274	0.14354	-0.21859	0.34408	0.06932	0.43711	0.662

Note. Confidence intervals computed with method: Standard (Delta method)

Note. Betas are completely standardized effect sizes

[5]

Conditional Mediation

Moderator levels					95% C.I. (a)				
treatment_seeking	Type	Effect	Estimate	SE	Lower	Upper	β	z	p
no		perscon ⇒ UCL_Avoidant	-0.16163	0.16586	-0.48670	0.16344	-0.14920	-0.97451	0.330
no		perscon ⇒ AvoidanceBehavior	0.06335	0.77721	-1.45995	1.58666	0.01002	0.08151	0.935
no		treatcon ⇒ UCL_Depressive	0.21642	0.21248	-0.20004	0.63288	0.21946	1.01851	0.308
no		treatcon ⇒ UCL_Palliative	0.32089	0.23798	-0.14555	0.78733	0.33626	1.34838	0.178
no		treatcon ⇒ UCL_Avoidant	0.39151	0.27498	-0.14744	0.93046	0.34276	1.42379	0.155
no		treatcon ⇒ AvoidanceBehavior	-1.35904	1.28856	-3.88457	1.16650	-0.20379	-1.05469	0.292
no		illcoher ⇒ UCL_Depressive	0.07094	0.13429	-0.19227	0.33415	0.08870	0.52826	0.597
no		illcoher ⇒ UCL_Palliative	0.10295	0.15041	-0.19184	0.39774	0.13302	0.68448	0.494
no		illcoher ⇒ UCL_Avoidant	0.05613	0.17379	-0.28449	0.39675	0.06059	0.32299	0.747
no		illcoher ⇒ AvoidanceBehavior	1.96575	0.81438	0.36960	3.56190	0.36344	2.41381	0.016
no		emotrepr ⇒ UCL_Depressive	-0.17339	0.21259	-0.59006	0.24328	-0.22569	-0.81560	0.415
no		emotrepr ⇒ UCL_Palliative	0.19990	0.23810	-0.26677	0.66657	0.26887	0.83955	0.401
no		emotrepr ⇒ UCL_Avoidant	0.11867	0.27512	-0.42055	0.65789	0.13336	0.43135	0.666
no		emotrepr ⇒ AvoidanceBehavior	0.13814	1.28920	-2.38865	2.66494	0.02659	0.10715	0.915
no	Direct	timeline ⇒ IBSQoL	0.68306	0.57634	-0.44654	1.81266	0.16067	1.18517	0.236
no		timecycl ⇒ IBSQoL	-1.06275	0.75240	-2.53742	0.41192	-0.18953	-1.41248	0.158
no		conseque ⇒ IBSQoL	-0.31245	0.73628	-1.75553	1.13064	-0.08693	-0.42436	0.671
no		perscon ⇒ IBSQoL	-0.31406	0.46499	-1.22543	0.59732	-0.06457	-0.67540	0.499
no		treatcon ⇒ IBSQoL	0.09574	0.78361	-1.44009	1.63158	0.01867	0.12219	0.903
no		illcoher ⇒ IBSQoL	-0.70855	0.49636	-1.68140	0.26431	-0.17034	-1.42748	0.153
no		emotrepr ⇒ IBSQoL	1.10988	0.77242	-0.40403	2.62379	0.27778	1.43689	0.151
no	Total	timeline ⇒ IBSQoL	0.69246	0.64404	-0.56984	1.95476	0.16223	1.07518	0.282
no		timecycl ⇒ IBSQoL	-0.65626	0.86583	-2.35326	1.04073	-0.11657	-0.75796	0.448
no		conseque ⇒ IBSQoL	0.37105	0.82575	-1.24739	1.98948	0.10282	0.44935	0.653
no		perscon ⇒ IBSQoL	-0.31132	0.53797	-1.36573	0.74309	-0.06375	-0.57869	0.563
no		treatcon ⇒ IBSQoL	-0.38845	0.89192	-2.13658	1.35968	-0.07544	-0.43552	0.663
no		illcoher ⇒ IBSQoL	-0.22896	0.56370	-1.33379	0.87587	-0.05482	-0.40617	0.685
no		emotrepr ⇒ IBSQoL	1.11283	0.89236	-0.63618	2.86183	0.27740	1.24705	0.212
yes	Indirect	timeline ⇒ UCL_Depressive ⇒ IBSQoL	-0.00197	0.08308	-0.16481	0.16087	-4.61e−4	-0.02372	0.981
yes		timeline ⇒ UCL_Palliative ⇒ IBSQoL	0.00832	0.02044	-0.03174	0.04838	0.00195	0.40725	0.684
yes		timeline ⇒ UCL_Avoidant ⇒ IBSQoL	0.00840	0.02032	-0.03141	0.04822	0.00197	0.41359	0.679
yes		timeline ⇒ AvoidanceBehavior ⇒ IBSQoL	0.29951	0.13684	0.03130	0.56772	0.07010	2.18868	0.029

Note. Confidence intervals computed with method: Standard (Delta method)

Note. Betas are completely standardized effect sizes

[5]

Conditional Mediation

Moderator levels					95% C.I. (a)				
treatment_seeking	Type	Effect	Estimate	SE	Lower	Upper	β	z	p
yes		timecycl ⇒ UCL_Depressive ⇒ IBSQoL	-0.07013	0.09839	-0.26296	0.12271	-0.01244	-0.71275	0.476
yes		timecycl ⇒ UCL_Palliative ⇒ IBSQoL	-0.02416	0.03687	-0.09643	0.04810	-0.00429	-0.65535	0.512
yes		timecycl ⇒ UCL_Avoidant ⇒ IBSQoL	-0.02014	0.04300	-0.10442	0.06413	-0.00357	-0.46851	0.639
yes		timecycl ⇒ AvoidanceBehavior ⇒ IBSQoL	-0.01283	0.14677	-0.30051	0.27484	-0.00228	-0.08743	0.930
yes		conseque ⇒ UCL_Depressive ⇒ IBSQoL	0.14545	0.08697	-0.02501	0.31590	0.04027	1.67240	0.094
yes		conseque ⇒ UCL_Palliative ⇒ IBSQoL	-0.01076	0.02134	-0.05259	0.03106	-0.00298	-0.50440	0.614
yes		conseque ⇒ UCL_Avoidant ⇒ IBSQoL	-0.02592	0.05384	-0.13144	0.07960	-0.00718	-0.48142	0.630
yes		conseque ⇒ AvoidanceBehavior ⇒ IBSQoL	0.60588	0.15927	0.29373	0.91804	0.16774	3.80422	< .001
yes		perscon ⇒ UCL_Depressive ⇒ IBSQoL	-0.00317	0.09731	-0.19389	0.18755	-6.49e−4	-0.03258	0.974
yes		perscon ⇒ UCL_Palliative ⇒ IBSQoL	0.01363	0.02684	-0.03897	0.06624	0.00279	0.50802	0.611
yes		perscon ⇒ UCL_Avoidant ⇒ IBSQoL	-7.66e−4	0.01287	-0.02600	0.02447	-1.57e−4	-0.05953	0.953
yes		perscon ⇒ AvoidanceBehavior ⇒ IBSQoL	-0.17081	0.15089	-0.46656	0.12493	-0.03494	-1.13203	0.258
yes		treatcon ⇒ UCL_Depressive ⇒ IBSQoL	-0.11592	0.09671	-0.30547	0.07363	-0.02249	-1.19861	0.231
yes		treatcon ⇒ UCL_Palliative ⇒ IBSQoL	0.03277	0.04596	-0.05732	0.12286	0.00636	0.71297	0.476
yes		treatcon ⇒ UCL_Avoidant ⇒ IBSQoL	0.01529	0.03343	-0.05022	0.08080	0.00297	0.45747	0.647
yes		treatcon ⇒ AvoidanceBehavior ⇒ IBSQoL	-0.15142	0.14172	-0.42917	0.12634	-0.02938	-1.06845	0.285
yes		illcoher ⇒ UCL_Depressive ⇒ IBSQoL	-0.10744	0.09550	-0.29461	0.07973	-0.02570	-1.12507	0.261
yes		illcoher ⇒ UCL_Palliative ⇒ IBSQoL	0.00742	0.02136	-0.03444	0.04928	0.00178	0.34755	0.728
yes		illcoher ⇒ UCL_Avoidant ⇒ IBSQoL	-0.00220	0.01277	-0.02722	0.02283	-5.25e−4	-0.17196	0.863

Note. Confidence intervals computed with method: Standard (Delta method)

Note. Betas are completely standardized effect sizes

[5]

Conditional Mediation

Moderator levels					95% C.I. (a)				
treatment_seeking	Type	Effect	Estimate	SE	Lower	Upper	β	z	p
yes		illcoher ⇒ AvoidanceBehavior ⇒ IBSQoL	0.00432	0.13837	-0.26688	0.27553	0.00103	0.03125	0.975
yes		emotrepr ⇒ UCL_Depressive ⇒ IBSQoL	0.30384	0.11966	0.06932	0.53837	0.07567	2.53928	0.011
yes		emotrepr ⇒ UCL_Palliative ⇒ IBSQoL	0.01256	0.02441	-0.03530	0.06041	0.00313	0.51427	0.607
yes		emotrepr ⇒ UCL_Avoidant ⇒ IBSQoL	-0.00581	0.01653	-0.03821	0.02658	-0.00145	-0.35162	0.725
yes		emotrepr ⇒ AvoidanceBehavior ⇒ IBSQoL	0.25250	0.14053	-0.02293	0.52793	0.06288	1.79679	0.072
yes	Component	timeline ⇒ UCL_Depressive	-0.00176	0.07415	-0.14709	0.14357	-0.00215	-0.02372	0.981
yes		UCL_Depressive ⇒ IBSQoL	1.12047	0.30012	0.53225	1.70868	0.21439	3.73346	< .001
yes		timeline ⇒ UCL_Palliative	0.03956	0.08305	-0.12321	0.20233	0.05001	0.47631	0.634
yes		UCL_Palliative ⇒ IBSQoL	0.21042	0.26796	-0.31477	0.73562	0.03896	0.78527	0.432
yes		timeline ⇒ UCL_Avoidant	-0.07389	0.09596	-0.26197	0.11418	-0.07804	-0.77003	0.441
yes		UCL_Avoidant ⇒ IBSQoL	-0.11371	0.23191	-0.56824	0.34082	-0.02520	-0.49033	0.624
yes		timeline ⇒ AvoidanceBehavior	1.06676	0.44967	0.18543	1.94809	0.19297	2.37233	0.018
yes		AvoidanceBehavior ⇒ IBSQoL	0.28077	0.04949	0.18377	0.37776	0.36330	5.67326	< .001
yes		timecycl ⇒ UCL_Depressive	-0.06259	0.08619	-0.23152	0.10635	-0.05805	-0.72610	0.468
yes		timecycl ⇒ UCL_Palliative	-0.11483	0.09654	-0.30404	0.07438	-0.11006	-1.18951	0.234
yes		timecycl ⇒ UCL_Avoidant	0.17715	0.11155	-0.04147	0.39578	0.14186	1.58818	0.112
yes		timecycl ⇒ AvoidanceBehavior	-0.04570	0.52270	-1.07019	0.97878	-0.00627	-0.08744	0.930
yes		conseque ⇒ UCL_Depressive	0.12981	0.06939	-0.00620	0.26582	0.18782	1.87058	0.061
yes		conseque ⇒ UCL_Palliative	-0.05115	0.07772	-0.20348	0.10118	-0.07648	-0.65812	0.510
yes		conseque ⇒ UCL_Avoidant	0.22793	0.08980	0.05192	0.40395	0.28474	2.53812	0.011
yes		conseque ⇒ AvoidanceBehavior	2.15797	0.42083	1.33317	2.98278	0.46172	5.12793	< .001
yes		perscon ⇒ UCL_Depressive	-0.00283	0.08684	-0.17303	0.16737	-0.00303	-0.03258	0.974
yes		perscon ⇒ UCL_Palliative	0.06480	0.09726	-0.12583	0.25543	0.07159	0.66622	0.505
yes		perscon ⇒ UCL_Avoidant	0.00674	0.11238	-0.21352	0.22700	0.00622	0.05997	0.952
yes		perscon ⇒ AvoidanceBehavior	-0.60839	0.52662	-1.64055	0.42377	-0.09619	-1.15526	0.248

Note. Confidence intervals computed with method: Standard (Delta method)

Note. Betas are completely standardized effect sizes

[5]

Conditional Mediation

Moderator levels					95% C.I. (a)				
**treatment_seeking**	**Type**	**Effect**	**Estimate**	**SE**	**Lower**	**Upper**	**β**	**z**	**p**
yes		treatcon ⇒ UCL_Depressive	-0.10346	0.08174	-0.26367	0.05676	-0.10491	-1.26560	0.206
yes		treatcon ⇒ UCL_Palliative	0.15574	0.09155	-0.02370	0.33518	0.16320	1.70111	0.089
yes		treatcon ⇒ UCL_Avoidant	-0.13447	0.10579	-0.34181	0.07286	-0.11773	-1.27119	0.204
yes		treatcon ⇒ AvoidanceBehavior	-0.53930	0.49572	-1.51088	0.43228	-0.08087	-1.08792	0.277
yes		illcoher ⇒ UCL_Depressive	-0.09589	0.08127	-0.25517	0.06339	-0.11989	-1.17992	0.238
yes		illcoher ⇒ UCL_Palliative	0.03528	0.09102	-0.14312	0.21367	0.04558	0.38757	0.698
yes		illcoher ⇒ UCL_Avoidant	0.01931	0.10517	-0.18682	0.22544	0.02085	0.18363	0.854
yes		illcoher ⇒ AvoidanceBehavior	0.01540	0.49283	-0.95053	0.98133	0.00285	0.03125	0.975
yes		emotrepr ⇒ UCL_Depressive	0.27117	0.07829	0.11773	0.42462	0.35297	3.46385	< .001
yes		emotrepr ⇒ UCL_Palliative	0.05967	0.08768	-0.11219	0.23152	0.08026	0.68049	0.496
yes		emotrepr ⇒ UCL_Avoidant	0.05111	0.10131	-0.14746	0.24968	0.05744	0.50450	0.614
yes		emotrepr ⇒ AvoidanceBehavior	0.89933	0.47475	-0.03117	1.82983	0.17309	1.89430	0.058
yes	Direct	timeline ⇒ IBSQoL	0.58897	0.27386	0.05220	1.12573	0.13786	2.15059	0.032
yes		timecycl ⇒ IBSQoL	0.33754	0.31626	-0.28233	0.95740	0.05990	1.06727	0.286
yes		conseque ⇒ IBSQoL	0.90471	0.28071	0.35453	1.45489	0.25047	3.22295	0.001
yes		perscon ⇒ IBSQoL	-0.41493	0.31575	-1.03380	0.20394	-0.08488	-1.31409	0.189
yes		treatcon ⇒ IBSQoL	0.00253	0.30275	-0.59086	0.59591	4.90e-4	0.00834	0.993
yes		illcoher ⇒ IBSQoL	-0.26515	0.29528	-0.84390	0.31359	-0.06343	-0.89796	0.369
yes		emotrepr ⇒ IBSQoL	0.70381	0.29840	0.11895	1.28867	0.17528	2.35860	0.018
yes	Total	timeline ⇒ IBSQoL	0.90325	0.31125	0.29321	1.51329	0.21161	2.90199	0.004
yes		timecycl ⇒ IBSQoL	0.21026	0.36181	-0.49887	0.91939	0.03735	0.58114	0.561
yes		conseque ⇒ IBSQoL	1.61936	0.29129	1.04845	2.19028	0.44874	5.55930	< .001
yes		perscon ⇒ IBSQoL	-0.57604	0.36452	-1.29049	0.13840	-0.11795	-1.58028	0.114
yes		treatcon ⇒ IBSQoL	-0.21673	0.34313	-0.88925	0.45578	-0.04209	-0.63164	0.528
yes		illcoher ⇒ IBSQoL	-0.36307	0.34113	-1.03167	0.30552	-0.08694	-1.06434	0.287
yes		emotrepr ⇒ IBSQoL	1.26687	0.32862	0.62279	1.91095	0.31580	3.85517	< .001

Note. Confidence intervals computed with method: Standard (Delta method)

Note. Betas are completely standardized effect sizes

[5]

GLM Mediation Analysis

Mediators Models		
	m1	UCL_Depressive ~ treatment_seeking + timeline + conseque + emotrepr + timeline:treatment_seeking + conseque:treatment_seeking + emotrepr:treatment_seeking
	m2	AvoidanceBehavior ~ treatment_seeking + timeline + conseque + emotrepr + timeline:treatment_seeking + conseque:treatment_seeking + emotrepr:treatment_seeking
Full Model		
	m3	IBSQoL ~ UCL_Depressive + AvoidanceBehavior + treatment_seeking + timeline + conseque + emotrepr + UCL_Depressive:treatment_seeking + AvoidanceBehavior:treatment_seeking + timeline:treatment_seeking + conseque:treatment_seeking + emotrepr:treatment_seeking
Indirect Effects		
	IE 1	timeline ⇒ UCL_Depressive ⇒ IBSQoL
	IE 2	timeline ⇒ AvoidanceBehavior ⇒ IBSQoL
	IE 3	conseque ⇒ UCL_Depressive ⇒ IBSQoL
	IE 4	conseque ⇒ AvoidanceBehavior ⇒ IBSQoL
	IE 5	emotrepr ⇒ UCL_Depressive ⇒ IBSQoL
	IE 6	emotrepr ⇒ AvoidanceBehavior ⇒ IBSQoL

[3]

Path Model

Model Diagram

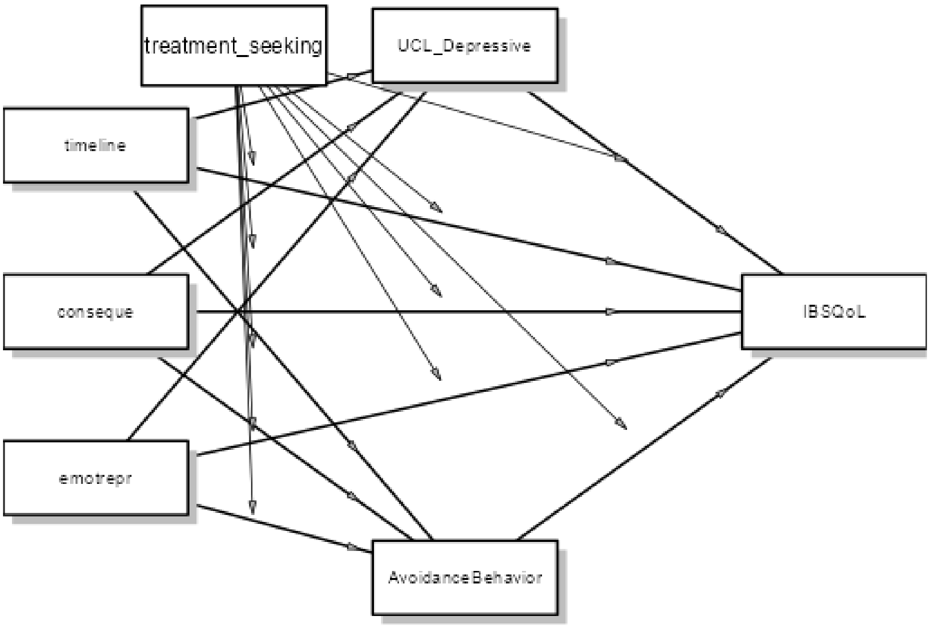

[4]

Model diagram notes
Moderators main effects are not shown
Covariances among IV are estimated but not shown

Mediation

Moderation effects (interactions)

Moderator	Interaction	Estimate	SE	Lower	Upper	β	z	p
	timeline:treatment_seeking1 ⇒ UCL_Depressive	0.2696	0.1524	-0.0291	0.568	0.16193	1.7690	0.077
	conseque:treatment_seeking1 ⇒ UCL_Depressive	-0.2500	0.1928	-0.6279	0.128	-0.18015	-1.2969	0.195
	emotrepr:treatment_seeking1 ⇒ UCL_Depressive	0.4427	0.2079	0.0352	0.850	0.29008	2.1291	0.033
	timeline:treatment_seeking1 ⇒ AvoidanceBehavior	0.1768	0.9378	-1.6613	2.015	0.01573	0.1885	0.850
	conseque:treatment_seeking1 ⇒ AvoidanceBehavior	0.4871	1.1864	-1.8382	2.813	0.05199	0.4106	0.681
	emotrepr:treatment_seeking1 ⇒ AvoidanceBehavior	0.2890	1.2797	-2.2192	2.797	0.02805	0.2259	0.821
	UCL_Depressive:treatment_seeking1 ⇒ IBSQoL	1.5868	0.6089	0.3935	2.780	0.48303	2.6062	0.009
	AvoidanceBehavior:treatment_seeking1 ⇒ IBSQoL	0.1500	0.0989	-0.0439	0.344	0.15906	1.5160	0.130
	timeline:treatment_seeking1 ⇒ IBSQoL	0.0911	0.5785	-1.0427	1.225	0.01071	0.1574	0.875
	conseque:treatment_seeking1 ⇒ IBSQoL	0.2651	0.7443	-1.1937	1.724	0.03740	0.3561	0.722
	emotrepr:treatment_seeking1 ⇒ IBSQoL	0.0342	0.7711	-1.4771	1.546	0.00439	0.0444	0.965

Conditional Mediation

Moderator levels					95% C.I. (a)				
treatment_seeking	Type	Effect	Estimate	SE	Lower	Upper	β	z	p
Average	Indirect	timeline ⇒ UCL_Depressive ⇒ IBSQoL	-0.0357	0.0429	-0.11975	0.0484	-0.00849	-0.831	0.406
Average		timeline ⇒ AvoidanceBehavior ⇒ IBSQoL	0.2505	0.1148	0.02560	0.4755	0.05962	2.183	0.029
Average		conseque ⇒ UCL_Depressive ⇒ IBSQoL	0.0812	0.0882	-0.09159	0.2540	0.02330	0.921	0.357
Average		conseque ⇒ AvoidanceBehavior ⇒ IBSQoL	0.4310	0.1601	0.11725	0.7448	0.12362	2.692	0.007
Average		emotrepr ⇒ UCL_Depressive ⇒ IBSQoL	0.0262	0.0406	-0.05336	0.1057	0.00675	0.645	0.519
Average		emotrepr ⇒ AvoidanceBehavior ⇒ IBSQoL	0.1594	0.1402	-0.11530	0.4342	0.04111	1.137	0.255
Average	Component	timeline ⇒ UCL_Depressive	-0.1221	0.0762	-0.27147	0.0272	-0.14839	-1.603	0.109
Average		UCL_Depressive ⇒ IBSQoL	0.2920	0.3004	-0.29674	0.8807	0.05718	0.972	0.331
Average		timeline ⇒ AvoidanceBehavior	1.1853	0.4689	0.26619	2.1043	0.21328	2.528	0.011
Average		AvoidanceBehavior ⇒ IBSQoL	0.2114	0.0488	0.11572	0.3070	0.27952	4.331	< .001
Average		conseque ⇒ UCL_Depressive	0.2782	0.0964	0.08928	0.4671	0.40740	2.886	0.004
Average		conseque ⇒ AvoidanceBehavior	2.0390	0.5932	0.87632	3.2017	0.44224	3.437	< .001
Average		emotrepr ⇒ UCL_Depressive	0.0897	0.1040	-0.11409	0.2935	0.11808	0.863	0.388
Average		emotrepr ⇒ AvoidanceBehavior	0.7542	0.6399	-0.49987	2.0084	0.14708	1.179	0.238
Average	Direct	timeline ⇒ IBSQoL	0.4869	0.2878	-0.07711	1.0509	0.11586	1.692	0.091
Average		conseque ⇒ IBSQoL	0.8071	0.3755	0.07112	1.5431	0.23148	2.149	0.032
Average		emotrepr ⇒ IBSQoL	0.8204	0.3859	0.06415	1.5767	0.21157	2.126	0.033
Average	Total	timeline ⇒ IBSQoL	0.8153	0.3205	0.18724	1.4434	0.18994	2.544	0.011
Average		conseque ⇒ IBSQoL	1.2384	0.4054	0.44382	2.0330	0.34773	3.055	0.002
Average		emotrepr ⇒ IBSQoL	1.1925	0.4373	0.33546	2.0496	0.30106	2.727	0.006
no	Indirect	timeline ⇒ UCL_Depressive ⇒ IBSQoL	0.1288	0.1032	-0.07335	0.3310	0.03004	1.249	0.212
no		timeline ⇒ AvoidanceBehavior ⇒ IBSQoL	0.1496	0.1265	-0.09830	0.3975	0.03488	1.183	0.237
no		conseque ⇒ UCL_Depressive ⇒ IBSQoL	-0.2022	0.1512	-0.49851	0.0941	-0.05682	-1.337	0.181
no		conseque ⇒ AvoidanceBehavior ⇒ IBSQoL	0.2449	0.1750	-0.09814	0.5879	0.06882	1.399	0.162
no		emotrepr ⇒ UCL_Depressive ⇒ IBSQoL	0.0660	0.1055	-0.14073	0.2728	0.01668	0.626	0.531

Note. Confidence intervals computed with method: Standard (Delta method)

Note. Betas are completely standardized effect sizes

[5]

Conditional Mediation

Moderator levels					95% C.I. (a)				
treatment_seeking	Type	Effect	Estimate	SE	Lower	Upper	β	z	p
no		emotrepr ⇒ AvoidanceBehavior ⇒ IBSQoL	0.0832	0.1664	-0.24296	0.4093	0.02101	0.500	0.617
no	Component	timeline ⇒ UCL_Depressive	-0.2569	0.1365	-0.52450	0.0106	-0.31216	-1.882	0.060
no		UCL_Depressive ⇒ IBSQoL	-0.5014	0.3004	-1.09015	0.0873	-0.09623	-1.669	0.095
no		timeline ⇒ AvoidanceBehavior	1.0969	0.8402	-0.54989	2.7436	0.19738	1.305	0.192
no		AvoidanceBehavior ⇒ IBSQoL	0.1364	0.0488	0.04074	0.2320	0.17672	2.795	0.005
no		conseque ⇒ UCL_Depressive	0.4032	0.1805	0.04947	0.7569	0.59046	2.234	0.025
no		conseque ⇒ AvoidanceBehavior	1.7954	1.1107	-0.38160	3.9724	0.38941	1.616	0.106
no		emotrepr ⇒ UCL_Depressive	-0.1317	0.1950	-0.51393	0.2506	-0.17337	-0.675	0.500
no		emotrepr ⇒ AvoidanceBehavior	0.6097	1.2003	-1.74283	2.9623	0.11890	0.508	0.611
no	Direct	timeline ⇒ IBSQoL	0.4413	0.5043	-0.54705	1.4297	0.10290	0.875	0.381
no		conseque ⇒ IBSQoL	0.6746	0.6719	-0.64225	1.9914	0.18957	1.004	0.315
no		emotrepr ⇒ IBSQoL	0.8033	0.7096	-0.58738	2.1940	0.20298	1.132	0.258
no	Total	timeline ⇒ IBSQoL	0.7198	0.5742	-0.40561	1.8452	0.16768	1.254	0.210
no		conseque ⇒ IBSQoL	0.7173	0.7591	-0.77052	2.2050	0.20140	0.945	0.345
no		emotrepr ⇒ IBSQoL	0.9525	0.8203	-0.65523	2.5603	0.24047	1.161	0.246
yes	Indirect	timeline ⇒ UCL_Depressive ⇒ IBSQoL	0.0137	0.0736	-0.13049	0.1580	0.00319	0.187	0.852
yes		timeline ⇒ AvoidanceBehavior ⇒ IBSQoL	0.3647	0.1345	0.10103	0.6284	0.08459	2.711	0.007
yes		conseque ⇒ UCL_Depressive ⇒ IBSQoL	0.1663	0.0867	-0.00375	0.3363	0.04648	1.917	0.055
yes		conseque ⇒ AvoidanceBehavior ⇒ IBSQoL	0.6536	0.1633	0.33358	0.9737	0.18271	4.003	< .001
yes		emotrepr ⇒ UCL_Depressive ⇒ IBSQoL	0.3376	0.1219	0.09872	0.5765	0.08484	2.770	0.006
yes		emotrepr ⇒ AvoidanceBehavior ⇒ IBSQoL	0.2574	0.1344	-0.00614	0.5209	0.06468	1.914	0.056
yes	Component	timeline ⇒ UCL_Depressive	0.0127	0.0677	-0.12005	0.1454	0.01537	0.187	0.852
yes		UCL_Depressive ⇒ IBSQoL	1.0854	0.3004	0.49666	1.6741	0.20717	3.614	< .001
yes		timeline ⇒ AvoidanceBehavior	1.2737	0.4167	0.45697	2.0903	0.22919	3.057	0.002
yes		AvoidanceBehavior ⇒ IBSQoL	0.2864	0.0488	0.19071	0.3820	0.36907	5.868	< .001
yes		conseque ⇒ UCL_Depressive	0.1532	0.0678	0.02040	0.2860	0.22434	2.261	0.024
yes		conseque ⇒ AvoidanceBehavior	2.2826	0.4170	1.46532	3.0998	0.49507	5.474	< .001

Note. Confidence intervals computed with method: Standard (Delta method)

Note. Betas are completely standardized effect sizes

[5]

Conditional Mediation

Moderator levels					95% C.I. (a)				
treatment_seeking	Type	Effect	Estimate	SE	Lower	Upper	β	z	p
yes		emotrepr ⇒ UCL_Depressive	0.3110	0.0721	0.16970	0.4524	0.40953	4.313	< .001
yes		emotrepr ⇒ AvoidanceBehavior	0.8988	0.4438	0.02891	1.7686	0.17526	2.025	0.043
yes	Direct	timeline ⇒ IBSQoL	0.5324	0.2535	0.03559	1.0293	0.12348	2.100	0.036
yes		conseque ⇒ IBSQoL	0.9396	0.2738	0.40290	1.4763	0.26265	3.431	< .001
yes		emotrepr ⇒ IBSQoL	0.8376	0.2813	0.28616	1.3890	0.21050	2.977	0.003
yes	Total	timeline ⇒ IBSQoL	0.9109	0.2848	0.35276	1.4690	0.21220	3.199	0.001
yes		conseque ⇒ IBSQoL	1.7595	0.2850	1.20103	2.3180	0.49406	6.175	< .001
yes		emotrepr ⇒ IBSQoL	1.4325	0.3033	0.83807	2.0270	0.36165	4.723	< .001

Note. Confidence intervals computed with method: Standard (Delta method)

Note. Betas are completely standardized effect sizes

[5]

References

[1] The jamovi project (2021). *jamovi*. (Version 2.2) [Computer Software]. Retrieved from https://www.jamovi.org.

[2] R Core Team (2021). *R: A Language and environment for statistical computing*. (Version 4.0) [Computer software]. Retrieved from https://cran.r-project.org. (R packages retrieved from MRAN snapshot 2021-04-01).

[3] Gallucci, M. (2020). *jAMM: jamovi Advanced Mediation Models*. [jamovi module]. Retrieved from https://jamovi-amm.github.io/.

[4] Soetaert, K. (2019). *diagram: Functions for Visualising Simple Graphs (Networks), Plotting Flow Diagrams*. [R package]. Retrieved from https://cran.r-project.org/package=diagram.

[5] Rosseel, Y. (2019). lavaan: An R Package for Structural Equation Modeling. *Journal of Statistical Software, 48(2), 1-36. link*.

Results

One-Way ANOVA

One-Way ANOVA (Welch's)

	F	df1	df2	p
IBSQoL_NoAvoid	2.49	3	24.0	0.085

Group Descriptives

	Marital_status	N	Mean	SD	SE
IBSQoL_NoAvoid	single	72	31.7	23.1	2.73
	married	82	32.1	18.8	2.07
	in a relationship	89	31.5	17.2	1.82
	other	6	53.2	18.6	7.59

Plots

IBSQoL_NoAvoid

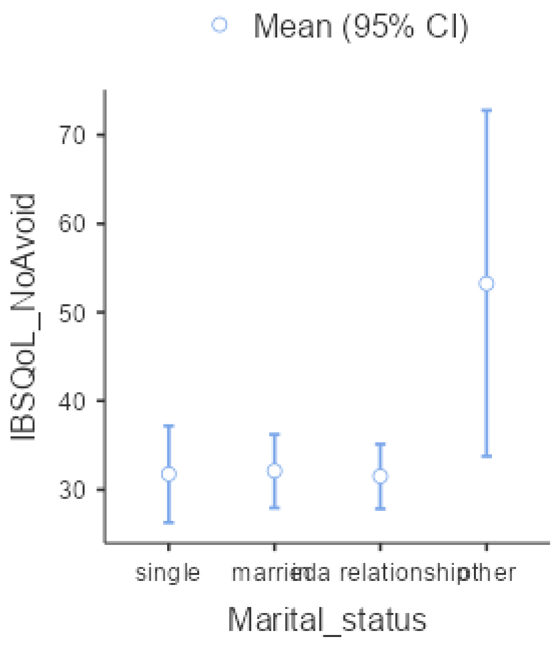

Correlation Matrix

Correlation Matrix

		Age	IBSQoL_NoAvoid
Age	Pearson's r	—	
	p-value	—	
IBSQoL_NoAvoid	Pearson's r	-0.095	—
	p-value	0.138	—

Note. * p < .05, ** p < .01, *** p < .001

Independent Samples T-Test

Independent Samples T-Test

		Statistic	df	p
IBSQoL_NoAvoid	Student's t	-1.22	247	0.223

Independent Samples T-Test

Independent Samples T-Test

		Statistic	df	p
IBSQoL_NoAvoid	Student's t	0.947	244	0.345

References

[1] The jamovi project (2021). *jamovi*. (Version 2.2) [Computer Software]. Retrieved from https://www.jamovi.org.

[2] R Core Team (2021). *R: A Language and environment for statistical computing*. (Version 4.0) [Computer software]. Retrieved from https://cran.r-project.org. (R packages retrieved from MRAN snapshot 2021-04-01).
